# RNA sequencing reveals upregulation of a transcriptomic program associated with stemness in metastatic prostate cancer cells selected for taxane resistance

**DOI:** 10.18632/oncotarget.25744

**Published:** 2018-07-13

**Authors:** Christina K. Cajigas-Du Ross, Shannalee R. Martinez, Leanne Woods-Burnham, Alfonso M. Durán, Sourav Roy, Anamika Basu, Joshua A. Ramirez, Greisha L. Ortiz-Hernández, Leslimar Ríos-Colón, Evgeny Chirshev, Evelyn S. Sanchez-Hernandez, Ubaldo Soto, Celine Greco, Claude Boucheix, Xin Chen, Juli Unternaehrer, Charles Wang, Carlos A. Casiano

**Affiliations:** ^1^ Center for Health Disparities and Molecular Medicine, Loma Linda University School of Medicine, Loma Linda, CA, USA; ^2^ Department of Entomology and Institute for Integrative Genome Biology, University of California Riverside, Riverside, CA, USA; ^3^ Department of Basic Sciences, Loma Linda University School of Medicine, Loma Linda, CA, USA; ^4^ Inserm, UMR-s935, Villejuif, France; ^5^ University of Paris XI, Université Paris-Saclay, Orsay, France; ^6^ Center for Genomics, Department of Basic Sciences, School of Medicine, Loma Linda University, Loma Linda, CA, USA; ^7^ Department of Medicine, Loma Linda University School of Medicine, Loma Linda, CA, USA

**Keywords:** prostate cancer, RNA sequencing, docetaxel resistance, cancer stem cells, therapeutic targeting

## Abstract

Patients with metastatic castration-resistant prostate cancer (mCRPC) develop resistance to conventional therapies including docetaxel (DTX). Identifying molecular pathways underlying DTX resistance is critical for developing novel combinatorial therapies to prevent or reverse this resistance. To identify transcriptomic signatures associated with acquisition of chemoresistance we profiled gene expression in DTX-sensitive and -resistant mCRPC cells using RNA sequencing (RNA-seq). PC3 and DU145 cells were selected for DTX resistance and this phenotype was validated by immunoblotting using DTX resistance markers (e.g. clusterin, ABCB1/P-gp, and LEDGF/p75). Overlapping genes differentially regulated in the DTX-sensitive and -resistant cells were ranked by Gene Set Enrichment Analysis (GSEA) and validated to correlate transcript with protein expression. GSEA revealed that genes associated with cancer stem cells (CSC) (e.g., *NES, TSPAN8, DPPP, DNAJC12, and MYC*) were highly ranked and comprised 70% of the top 25 genes differentially upregulated in the DTX-resistant cells. Established markers of epithelial-to-mesenchymal transition (EMT) and CSCs were used to evaluate the stemness of adherent DTX-resistant cells (2D cultures) and tumorspheres (3D cultures). Increased formation and frequency of cells expressing CSC markers were detected in DTX-resistant cells. DU145-DR cells showed a 2-fold increase in tumorsphere formation and increased DTX resistance compared to DU145-DR 2D cultures. These results demonstrate the induction of a transcriptomic program associated with stemness in mCRPC cells selected for DTX resistance, and strengthen the emerging body of evidence implicating CSCs in this process. In addition, they provide additional candidate genes and molecular pathways for potential therapeutic targeting to overcome DTX resistance.

## INTRODUCTION

Prostate cancer (PCa) is the most commonly diagnosed cancer and the second cause of cancer deaths among American men [1]. For men diagnosed with advanced PCa, treatment with curative intent is no longer an option, and androgen deprivation therapy (ADT) remains the main therapeutic modality [2, 3]. Despite its initial effectiveness at reducing tumor growth, ADT ultimately fails, resulting in metastatic castration-resistant prostate cancer (mCRPC), a lethal stage of the disease that is marked by recurrence of elevated prostate specific antigen (PSA) and progression of metastatic lesions [3, 4]. Since 2004, the standard first-line chemotherapeutic agent for the treatment of mCRPC has been the taxane drug docetaxel (DTX), a microtubule-stabilizing agent that moderately increases overall survival [4, 5]. Eventually, however, chemoresistance occurs in all DTX-treated patients resulting in continued disease progression [6]. In recent years, new treatment options for mCRPC have been developed, including the next-generation androgen receptor-targeting agents abiraterone acetate and enzalutamide, therapeutic vaccines, and the second generation taxane cabazitaxel [6, 7]. Unfortunately, these novel therapeutic agents, which are often administered sequentially or in combination with DTX, only moderately improve overall patient survival due to the development of therapy resistance.

Understanding the molecular mechanisms underlying the acquisition of PCa chemoresistance is critical for developing novel and effective combinatorial therapies for the prevention or reversal of taxane resistance. Several mechanisms involved in the development of DTX-resistance have been identified, including the increased expression and activity of multidrug resistance pumps (e.g. ABCB1/P-gp/MDR1), impaired apoptotic pathways (e.g. Bcl-2), cytokine and chemokine induction (e.g. IL-6, CCL2), alterations in microtubule structure and function, NF-kB pathway activation, and upregulation of stress survival proteins (e.g. Hsp27, clusterin, and LEDGF/p75) [2, 8, 9]. Unfortunately, efforts aimed at targeting or disrupting some of these pathways in the clinical setting have been largely unsuccessful [4]. This is illustrated by the recent failure of phase III clinical trials with Custirsen, a second generation oligonucleotide administered in combination with DTX designed to disrupt the production of clusterin (CLU), a cytoprotective anti-apoptotic chaperone protein overexpressed in PCa [10, 11]. Such failures highlight the importance of continued efforts towards discovering new mechanisms and molecular pathways associated with the taxane-resistant phenotype.

Chemoresistance, the primary cause of treatment failure, is driven by the survival of subpopulations of prostate tumor cells that eventually contribute to aggressive disease progression, characterized by metastasis to the bone and vital organs [12]. In addition, PCa tumors are highly heterogeneous, with many areas containing genetically distinct clones [13], a characteristic that also contributes to therapy resistance and tumor relapse [12]. An emerging explanation for both the development of resistance and the cellular heterogeneity in PCa and other solid tumors is the cancer stem cell (CSC) hypothesis, which proposes that solid tumors are organized hierachically with only a minor subset of cells capable of tumor-initiating and tumor-propagating capacity [12, 14].

Recent studies revealed that markers associated with epithelial-to-mesenchymal (EMT) transition and CSCs are elevated in DTX-resistant mCRPC cells [15, 16], and that CSCs derived from immortalized normal prostate epithelial cells showed increased DTX resistance compared to parental adherent cells [17]. Given the scarcity of next generation sequencing (NGS) studies examining the transcriptomic programs activated during development of DTX-resistance in mCRPC, we performed an RNA sequencing (RNA-seq) analysis on DTX-sensitive and DTX-resistant mCRPC cells in an effort to identify gene pathways potentially involved in taxane resistance. GSEA analysis of the overlapping upregulated genes in DTX-resistant PC3-DR and DU145-DR cells revealed an induction of a transcriptomic program associated with stemness as cells transitioned from DTX sensitivity to resistance. To validate this finding, we characterized the CSC phenotype in tumorspheres from DTX-resistant PC3-DR and DU145-DR cells using CSC markers previously validated in prostate tumorspheres. Understanding the role of CSCs in PCa chemoresistance, including the transcriptomic pathways that define their activation and maintenance is critical to identifying new targets for combinatorial therapies aimed at circumventing taxane resistance in mCRPC.

## RESULTS

### PC3-DR and DU145-DR cells upregulate markers of taxane resistance

**Figure 1:**
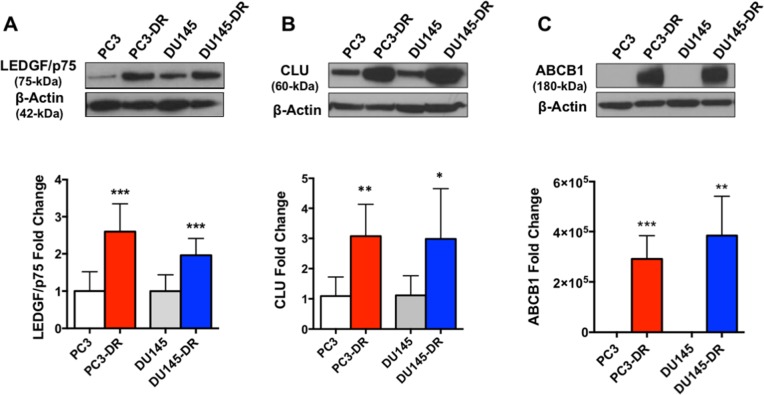
DTX-resistant PC3 and DU145 cell lines upregulate known markers of DTX resistance Upper panel: Western blots of **(A)** LEDGF/p75, **(B)** CLU, and **(C)** ABCB1 showing upregulation of these proteins in DTX-resistant PC3 and DU145 mCRPC cells, compared to the parental, sensitive cells. Bottom panel: quantification of fold change in protein expression **(A)** n=8; **(B)** n=5; **(C)** n=4 independent experiments. ^*^P <0.05; ^**^P < 0.05; ^***^P < 0.001. Due to the absence of ABCB1 expression in the parental PC3 and DU145 cells, for quantification purposes we normalized its expression in these cells to an arbitrary value of 0.10. Error bars represent mean ± standard deviation (SD).

We developed PC3-DR and DU145-DR cell lines by selecting and expanding the surviving cells in the presence of incrementally increasing concentrations of DTX until cells could be maintained in 10 nM DTX with minimal cell death [8, 18]. Our group reported recently that these DTX-resistant cell lines are also resistant to paclitaxel and cabazitaxel, other clinically relevant taxanes [8]. We also demonstrated that these DTX-resistant cells overexpress the stress oncoprotein Lens Epithelium Derived Growth Factor of 75 kD (LEDGF/p75), and that depletion of this protein partially resensitized these cells to DTX [8, 18]. In the present study, we confirmed that the DTX-resistant PC3-DR and DU145-DR cell lines used in the RNA-seq analysis and other experiments displayed significant upregulation of proteins previously implicated by our group and others in PCa progression and DTX resistance [8, 9, 19–23] including LEDGF/p75, CLU, and ATP-binding cassette sub-family B member 1 (ABCB1), compared to the sensitive cells (Figure 1A-1C). This validation step was critical prior to initiating our RNA-seq analysis comparing the transcriptome profiles of DTX-sensitive and DTX-resistant PCa cell lines.

### RNA-seq analysis revealed upregulation of genes associated with CSC-like characteristics

**Figure 2:**
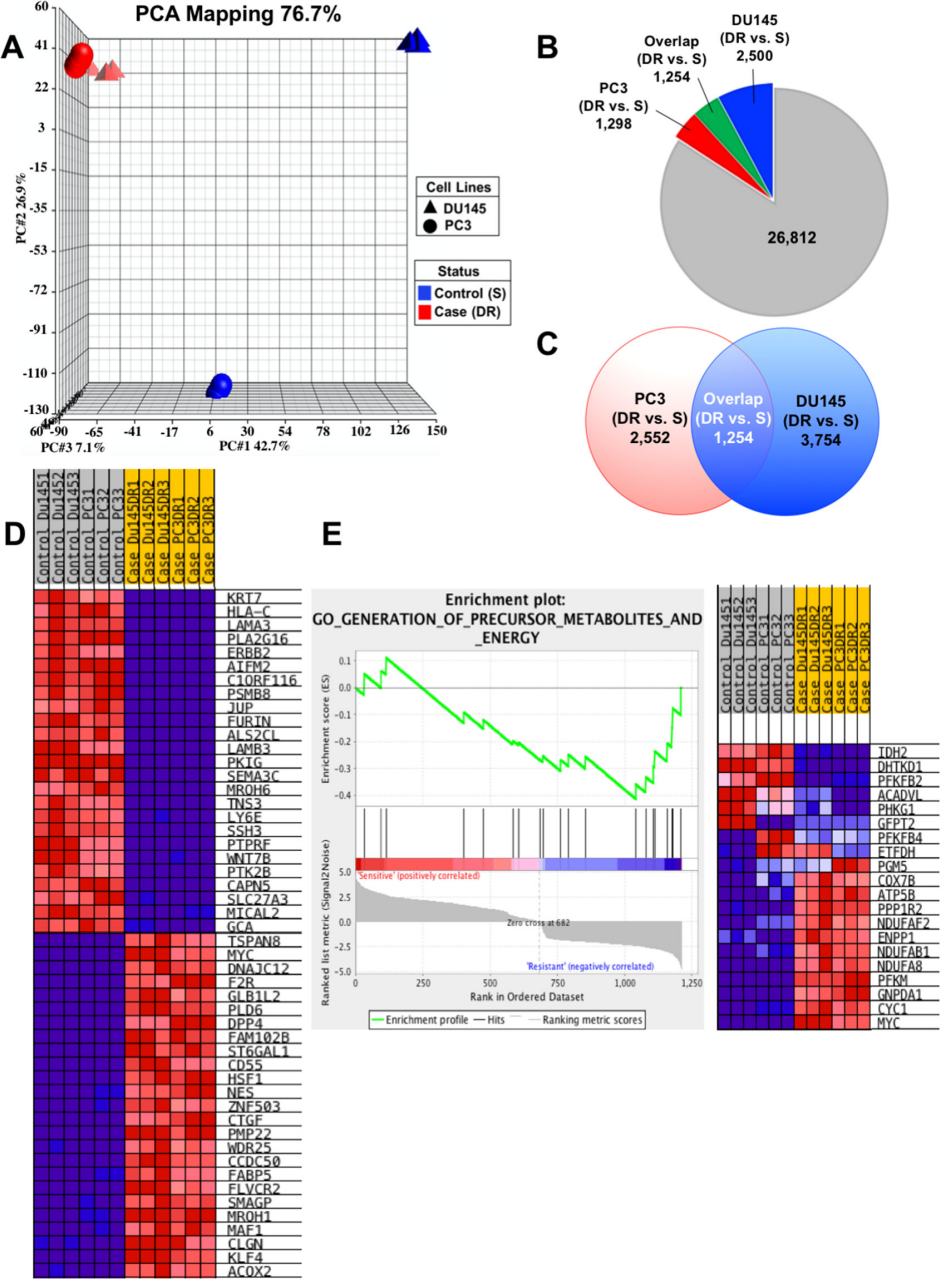
Gene expression profiling analysis reveals upregulation of CSC-associated genes **(A)** Principal component Analysis (PCA) mapping demonstrates clustering of DTX-resistant cell lines based on gene expression profiles. **(B)** Diagram showing the distribution of statistically significant differentially regulated genes in each cell line, comparing DTX-resistant (DR) to sensitive (S). **(C)** Diagram demonstrating the overlap or shared genes common to both PC3 and DU145 cells, comparing DR to S. **(D)** Heatmap of the top ranked genes generated using GSEA analysis on the common overlap genes between both sensitive PC3 and DU145 cells compared to PC3-DR and DU145-DR. Red represents fold upregulation and blue represents fold downregulation. **(E)** GSEA gene set pathway analysis revealed one pathway to be significantly enriched in the DTX-resistant PC3-DR and DU145-DR cells compared to sensitive PC3 and DU145 cells (*P*= 0.032) involving precursor metabolites and energy. A positive value indicates correlation with the sensitive phenotype and negative value indicates correlation with the resistant phenotype.

Principal Component Analysis (PCA) 3D mapping of our RNA-seq data demonstrated that the DTX-sensitive PC3 and DU145 cells were clearly separated from each other based on global transcriptome expression profiles (Figure 2A). However, once these cell lines became DTX-resistant they were clustered together spatially, suggesting an acquired similarity in transcriptomic profiles. Global gene heat map also demonstrated the clustering of the DTX-resistant cell lines based on their transcriptome expression profiles (See Supplementary Figure 1). Our RNA-seq data revealed that of 31,864 total genes detected, 3,754 and 2,552 were differentially upregulated with statistical significance (FDR > 0.05, and fold change [FC] > 2) in the DU145-DR and PC3-DR cells, respectively, compared to their DTX-sensitive counterparts (Figure 2B, 2C). Of these genes, 1,254 overlapped between the PC3-DR and DU145-DR cells. GSEA of the top 25 ranked overlap genes between the DTX-sensitive and DTX-resistant PC3 and DU145 cells revealed a distinct on/off switch of genes, suggesting a pattern of upregulated/downregulated genes associated with the development of DTX-resistance in both cell lines (Figure 2D) (see Supplementary Figure 2 for top 50 ranked genes). An exhaustive PubMed literature search also revealed that 17 of the top 25 (70%) ranked overlapping genes upregulated in the DTX-resistant cell lines have been shown to be associated with or contribute to a CSC phenotype (Table 1). Top downregulated genes are listed in Supplementary Table 1.

**Table 1:** GSEA top-ranked RNA-seq upregulated genes

Gene Name	Gene Title	Rank Score (GSEA)	Log_2_ Fold Change PC3 vs. PC3-DR	Log_2_ Fold Change DU145 vs. DU145-DR	Stem Cell-Associated
TSPAN8	tetraspanin 8	-4.782	5.357	5.815	YES
MYC	v-myc myelocytomatosis viral oncogene homolog	-4.497	3.813	4.727	YES
DNAJC12	DNAJ (Hsp40) homolog, subfamily C, member 12	-4.466	4.411	6.032	YES
F2R	coagulation factor II (thrombin) receptor	-4.429	4.289	3.820	YES
GLB1L2	galactosidase beta 1-like 2	-4.206	3.421	3.670	YES
PLD6	phospholipase D family member 6	-4.194	3.537	3.529	YES
DPP4	dipeptidyl-peptidase 4 (CD26, adenosine deaminase complexing protein 2)	-4.135	3.890	4.956	YES
FAM102B	family with sequence similarity 102, member B	-3.967	3.196	3.083	YES
ST6GAL1	ST6 beta-galactosamide alpha-2,6-sialyltransferase 1	-3.909	2.925	1.439	YES
CD55	CD55 molecule, decay accelerating factor for complement (Cromer blood group)	-3.908	2.652	3.861	YES
HSF1	heat shock transcription factor 1	-3.885	3.083	3.990	YES
NES	nestin	-3.806	3.477	5.980	YES
ZNF503	zinc finger protein 503	-3.725	2.409	3.432	YES
CTGF	connective tissue growth factor	-3.685	2.779	2.695	YES
PMP22	peripheral myelin protein 22	-3.641	2.798	2.597	YES
WDR25	WD repeat domain 25	-3.633	2.690	2.673	-
CCDC50	coiled-coil domain containing 50	-3.622	2.355	2.989	-
FABP5	fatty acid binding protein 5 (psoriasis-associated)	-3.591	2.538	4.071	YES
FLVCR2	feline leukemia virus subgroup C receptor 2	-3.586	2.460	3.167	-
SMAGP	small cell adhesion glycoprotein	-3.579	2.538	3.345	-
MROH1	maestro heat-like repeat family member 1	-3.548	2.535	3.679	-
MAF1	MAF1 homolog (S. cerevisiae)	-3.501	2.470	3.158	-
CLGN	calmegin	-3.455	2.784	2.488	YES
KLF4	kruppel-like factor 4 (gut)	-3.452	2.271	2.657	YES
ACOX2	acyl-coenzyme A oxidase 2, branched chain	-3.437	2.552	2.371	-

Gene Set Enrichment Analysis (GSEA) also identified the gene set “GO_Generation of Precursor Metabolites and Energy” as the only significantly enriched pathway in the DTX-resistant PC3-DR and DU145-DR cells (*P* = 0.032) (Figure 2E). This analysis yielded 8 genes (*NADUFAF2*, *ENPP1*, *NDUFAB1*, *NDUFA8*, *PFKM*, *GNPDA1*, *CYC1*, *MYC*) that were positive for core enrichment in this gene set. Of these genes, ectonucleotide phosphodiesterase 1 (*ENPP1*), cytochrome c-1 (*CYC1*), NADH dehydrogenase 1 alpha/beta subcomplex 1 (*NDUFAB1*), and v-myc myelocytomatosis viral oncogene homolog (*MYC*) have been associated with stem cell maintenance, phenotype acquisition, or reprogramming [24–29], suggesting that upregulation of specific genes involved in metabolism may contribute to an enrichment of cells with CSC-like characteristics (Figure 2E). Taken together, the RNA-seq analysis of transcript expression in DTX-sensitive vs. DTX-resistant PCa cell lines provides evidence for the acquisition of a transcriptomic program associated with stemness as a mechanism contributing to the development of DTX-resistance.

### Validation of transcript and protein expression of selected genes in DTX-resistant cells confirmed RNA-seq results

**Figure 3:**
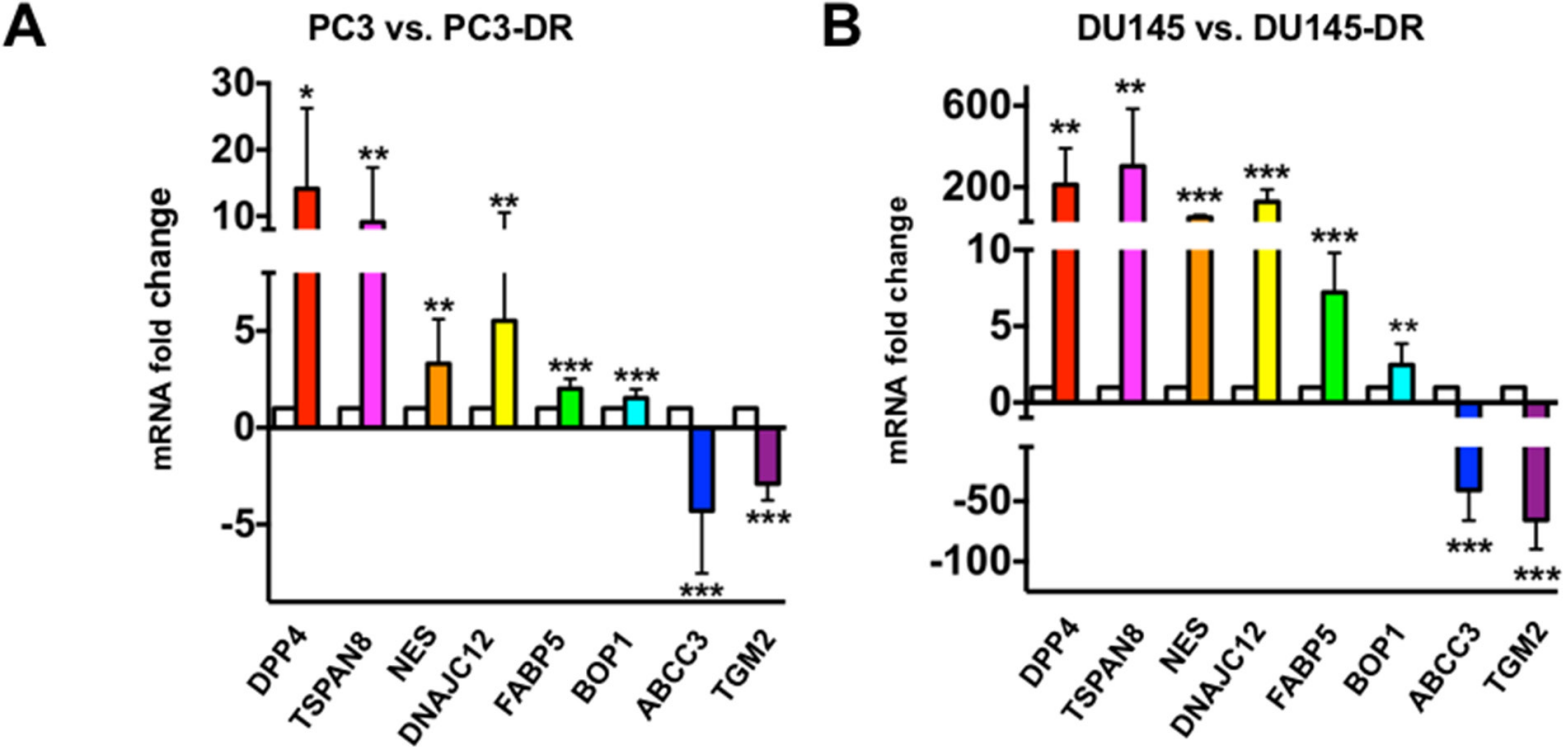
In-house qPCR validation of the expression of selected top-ranked genes from RNA-seq results in DTX-sensitive and DTX–resistant mCRPC cells qPCR validation for selected genes in **(A)** PC3 vs. PC3-DR and **(B)** DU145 vs. DU145-DR cells. White bars represent parental PC3 or DU145 and colored bars represent PC3-DR or DU145-DR. ^*^P <0.05; ^**^P < 0.05; ^***^P < 0.001. All RNA samples were analyzed in at least three independent experiments using at least three biological replicates per experiment. Error bars represent mean ± SD.

To confirm the RNA-seq data, we performed in-house qPCR validation on selected genes that showed robust upregulation in both PC3-DR and DU145-DR cells, compared to the sensitive, parental cell lines. The selection of specific genes for validation was determined by two criteria: the GSEA ranked gene order (Table 1 and Supplementary Table 1), and exhaustive literature searches implicating these genes in cancer, PCa, therapy resistance, DTX resistance, stem cells, CSCs, or EMT. For our in-house validation of RNA-seq data, new RNA samples were extracted from a different set of DTX-sensitive and DTX-resistant cells than those used for the RNA-seq analysis. Consistent with the RNA-seq results, transcript expression of dipeptidyl peptidase 4 (DPP4), tetraspanin 8 (TSPAN8), nestin (NES), DNAJ heath shock protein family member C12 (DNAJC12), fatty acid binding protein 5 (FABP5), and block of proliferation 1 (BOP1) were upregulated in PC3-DR and DU145-DR cells compared to the corresponding sensitive cell lines (Figure 3). As an internal control for in-house validation, we also chose two genes found robustly downregulated in the RNA-seq results, transglutaminase 2 (TGM2) and ATP-binding cassette subfamily C member 3 (ABCC3). Transcript expression of both genes was robustly downregulated in PC3-DR and DU145-DR cells compared to the sensitive cell lines, further confirming the RNA-seq results (Figure 3). The magnitude of fold-increase observed for each of these genes was more robust in DU145-DR cells than in PC3-DR cells, suggesting cell-type dependent differences in gene expression during the acquisition of resistance to DTX. Despite these differences, *P* values were consistently < 0.01 for each of the selected genes in both DTX-resistant cell lines.

**Figure 4:**
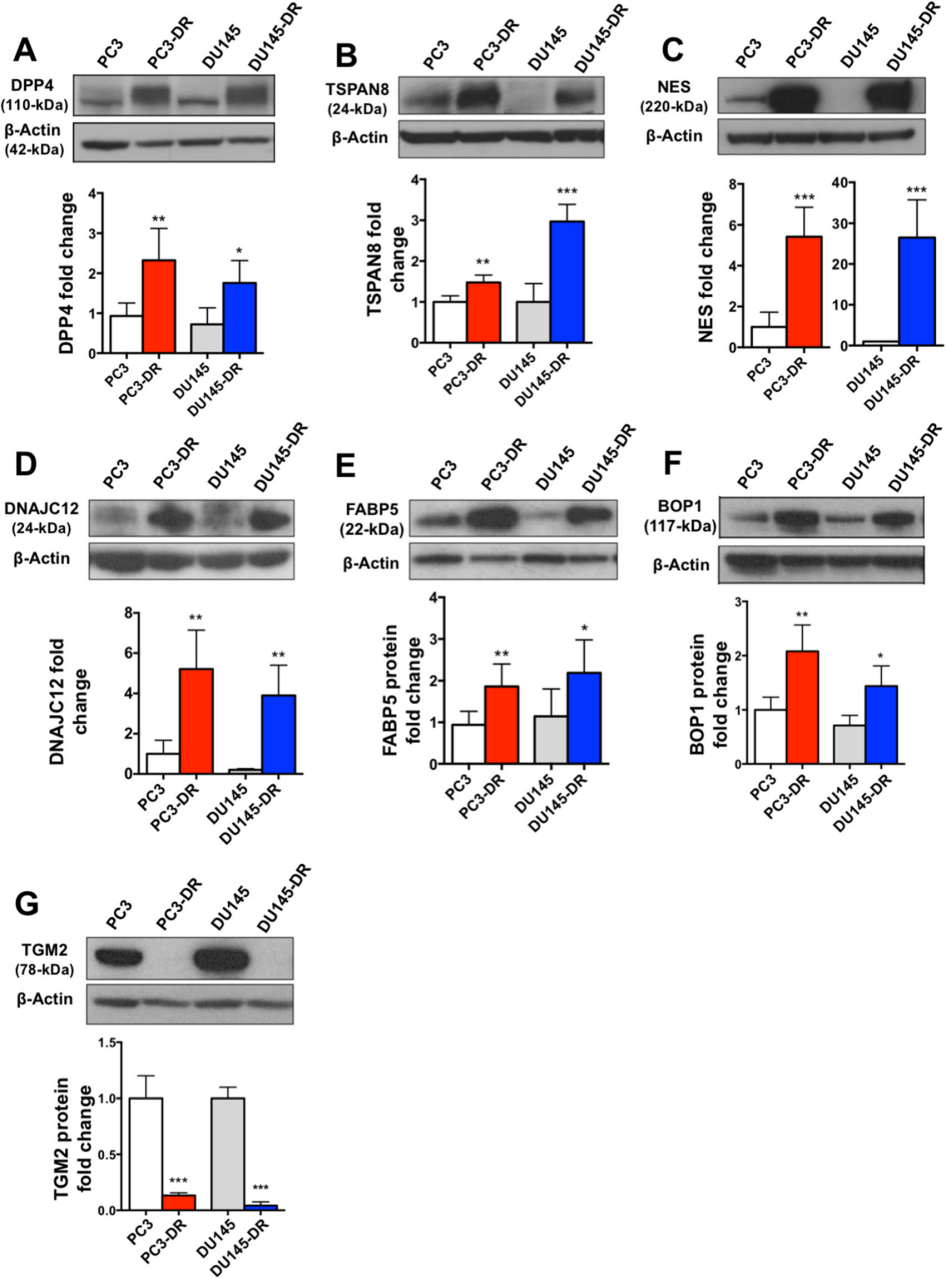
Protein expression validation of RNA-seq results in DTX-sensitive and DTX-resistant mCRPC cells Representative Western blot images and protein fold change quantification are shown for **(A)** DPP4 (n= 3), **(B)** TSPAN8 (n= 4), **(C)** NES (n= 6), **(D)** DNAJC12 (n= 4), **(E)** FABP5 (n= 7), **(F)** BOP1 (n=4), and **(G)** TGM2 (n=4). ^*^P< 0.05; ^**^P< 0.05; ^***^P< 0.001. All proteins were analyzed in at least three independent experiments. Error bars represent mean ± SD.

After validation of the transcript expression of selected genes in the DTX-resistant PC3-DR and DU145-DR cells, we sought to confirm corresponding protein upregulation in these cells compared to their sensitive counterparts by immunoblotting using specific antibodies. Significant upregulation of DPP4, TSPAN8, NES, DNAJC12, FABP5, and BOP1 was observed in the PC3-DR and DU145-DR cells, consistent with the qPCR and RNA-seq results (Figure 4A-4F). Also consistent with the RNA-seq and qPCR results, the protein expression of TGM2 was downregulated in the DTX-resistant cells (Figure 4G).

### Analysis of cancer gene microarray datasets reveals consistent upregulation of DNAJC12, FABP5, and BOP1 in PCa tissues

After confirming that transcript and protein expression of selected genes reflected the upregulation observed in the RNA-seq analysis, we sought to examine the expression of these genes in human PCa tissues. Transcript expression of the selected genes in PCa tissues, compared to normal prostate tissue, was analyzed using 16 PCa gene expression microarray datasets from the Oncomine database. All 16 datasets had data for FABP5, whereas data for DPP4 and TSPAN8 were available in 15 datasets, and data for BOP1, DNAJC12 and NES were available in 14, 10 and 8 datasets, respectively.

**Figure 5:**
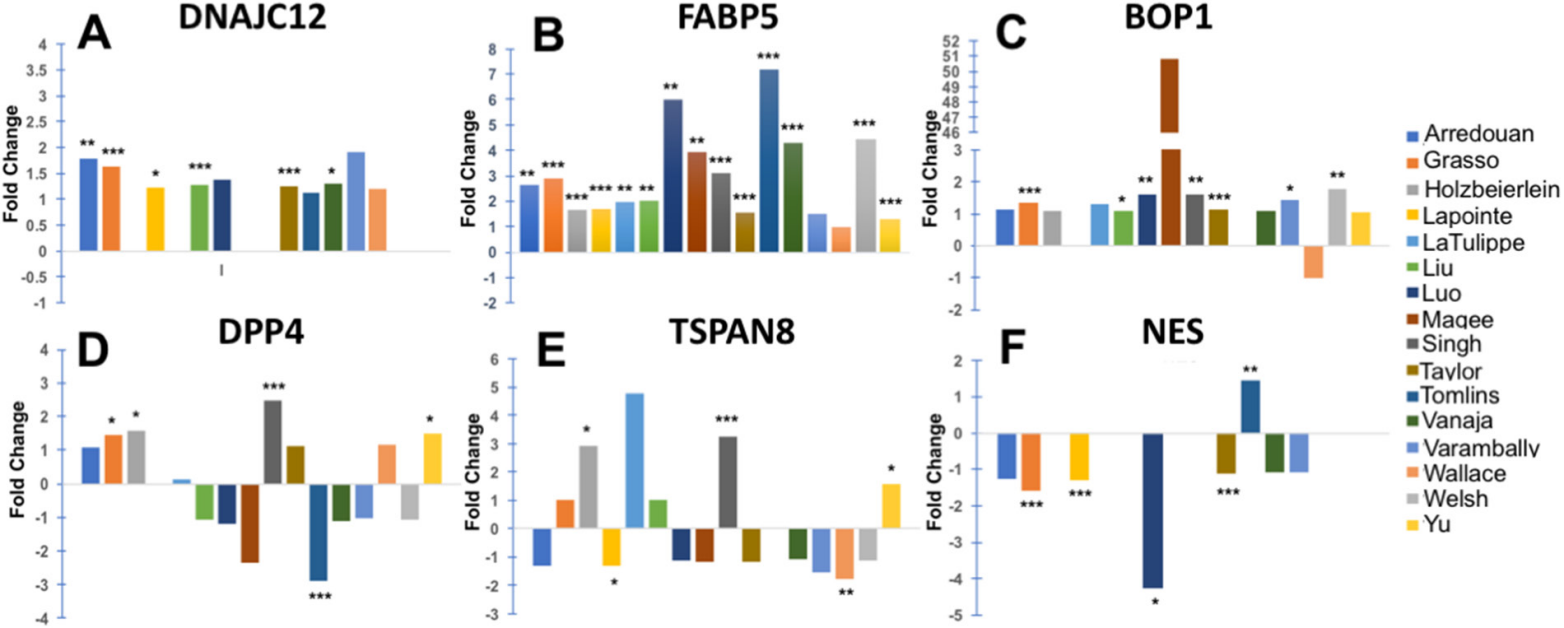
Expression of selected top-ranked genes in clinical PCa tissues Fold change between transcript expression levels of selected top ranked genes (from RNA-seq analysis) in prostate tumors versus normal prostate tissues as derived from cancer gene microarray datasets in the Oncomine database. Selected genes were **(A)** DNAJC12; **(B)** FABP5; **(C)** BOP1; **(D)** DPP4; **(E)** TSPAN8; and **(F)** NES. Individual dataset names appear in the legend box at the right. *P* values for the differences in gene expression between PCa and normal prostate tissues were obtained from Oncomine. The number of samples in each dataset is different, therefore higher fold change does not always correspond to statistical significance. ^*^P< 0.05, ^**^P< 0.01, ^***^P< 0.001.

Of the selected genes, DNAJC12, FABP5, and BOP1 were the most consistently upregulated in prostate tumors compared to normal prostate tissues in the dataset collection (Figure 5A-5C), with significant upregulation of DNAJC12 in 6 of the 14 datasets (Figure 5A), FABP5 in 14 of the 16 data sets (Figure 5B), and BOP1 in 7 of the 14 datasets (Figure 5C). DPP4 and TSPAN8 transcripts were significantly upregulated only in 4 of the 14 datasets (Figure 5D-5E). Interestingly, significant upregulation of NES transcript was detected only in 1 of the 8 datasets (Figure 5F). The magnitude of the fold-increase observed for the individual genes was modest, with only FABP5 showing over 2-fold increase in multiple datasets. However, the *P* values were <0.01 for most of the DNAJC12, FABP5, and BOP1 datasets, indicating that upregulation of these transcripts is highly significant in PCa tissues compared to normal tissues. On the other hand, NES transcripts were significantly downregulated in 4 of the 8 datasets, whereas DPP4 and TSPAN8 transcripts displayed significant downregulation in 1 and 2 out of 15 datasets, respectively.

### PC3-DR and DU145-DR cells upregulate markers associated with EMT and CSCs

**Figure 6:**
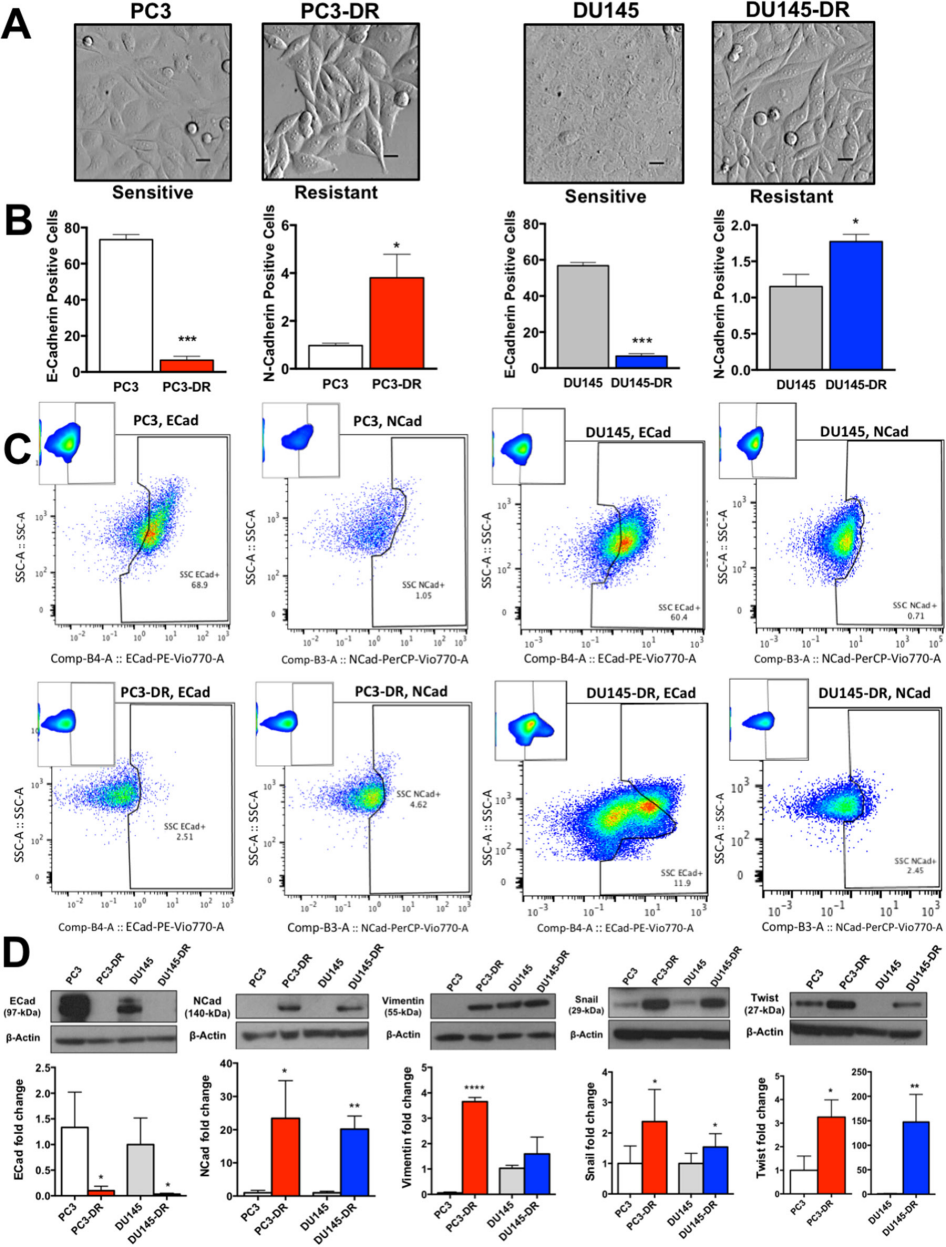
DTX-resistant mCRPC cells exhibit a mesenchymal-like phenotype compared to DTX-sensitive cells **(A)** Differences in morphology between DTX-sensitive and DTX-resistant PC3 and DU145 cells visualized by Hoffman modulation contrast microscopy (scale bar set at 40 μm). **(B)** Percent of live PC3 and DU145 cells (DTX sensitive and -resistant) that stained positive for E-cadherin and N-cadherin as determined by flow cytometry (n=3 biological replicates) ^*^P< 0.05, ^**^P< 0.01, ^***^P< 0.001. **(C)** Representative flow charts of bar graph data showing downregulation of E-cadherin and upregulation of N-cadherin in the DTX-resistant cell lines. **(D)** Representative Western blots showing expression (upper panels) and quantification (lower panels) of E-cadherin (n=3), N-cadherin (n=3), Vimentin (n=3), Snail (n=3), and Twist (n=3). ^*^P< 0.05, ^**^P< 0.01, ^***^P< 0.001. Error bars represent mean ± SEM.

**Figure 7:**
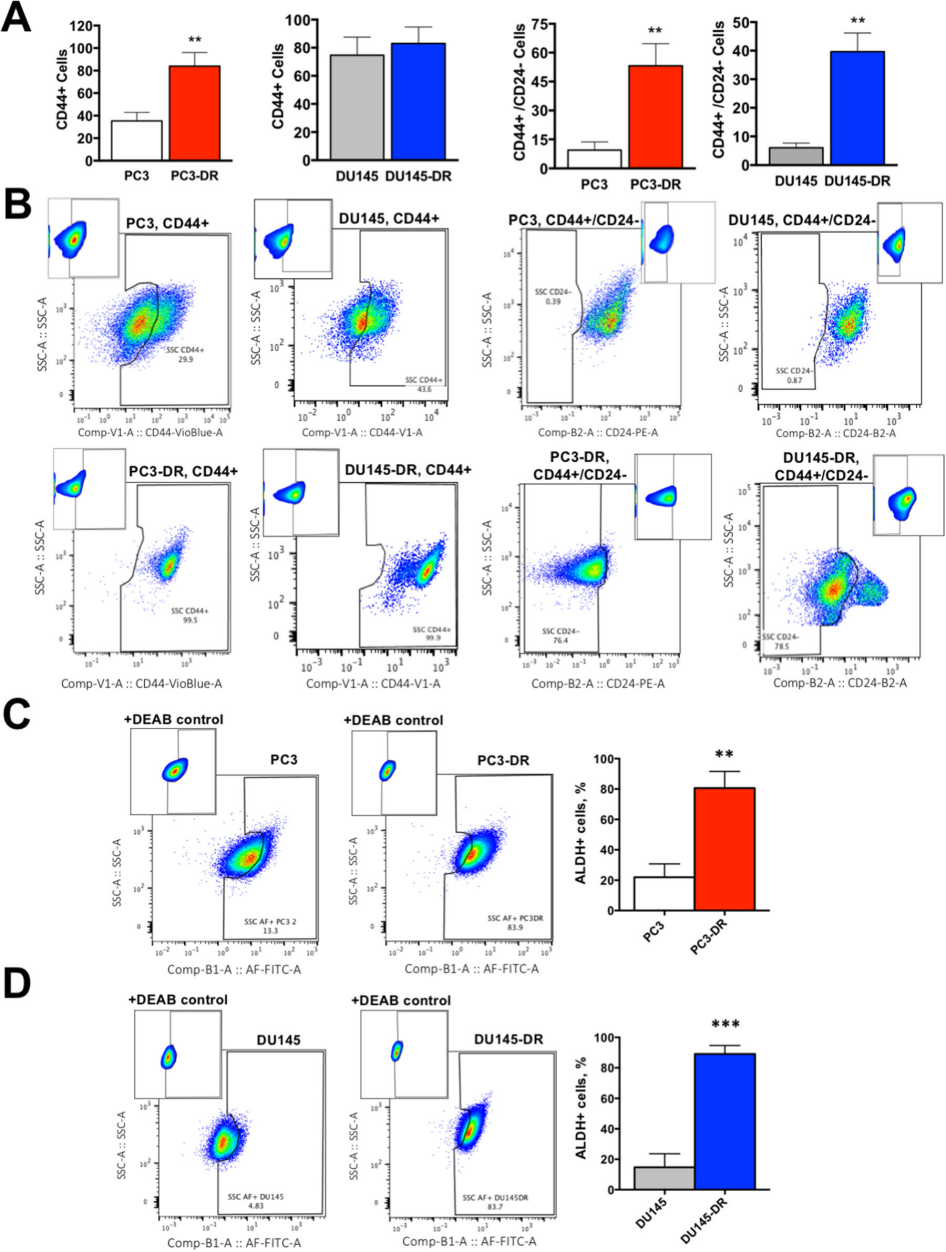
DTX-resistant mCRPC cells upregulate markers associated with CSC-like characteristics compared to DTX-sensitive cells **(A)** Percent of CD44+ and CD44+/CD24- cells for PC3 vs PC3-DR and DU145 vs DU145-DR, with **(B)** representative flow cytometry plots showing compensation windows used in the FMO analysis for each marker. Flow data is represented as frequency of live cells determined by annexin-V staining. **(C)** PC3-DR and **(D)** DU145-DR cells have significantly greater ALDH activity (Aldefluor+) compared with sensitive PC3 and DU145 cells as determined by aldefluor assay (+DEAB control used for gating). Representative flow plots are shown together with bar graphs. All flow measurements were acquired from at least 3 independent experiments conducted separately. ^**^P< 0.01, ^***^P< 0.001. Error bars represent mean ± SEM.

The observation that highly ranked genes (GSEA) in the RNA-seq results were associated with CSC development or are known markers of CSCs (e.g. *NES*, *DPP4*, *TSPAN8*), led us to assess the expression of established EMT and CSC markers in our DTX-resistant cell lines. Microscopic assessment of DTX-resistant cells revealed a mesenchymal phenotype with clearly defined edges and the classical spindle-shaped morphology, compared to the flattened, polygonal-shaped sensitive PC3 and DU145 (Figure 6A). Using multicolor flow cytometry, we analyzed the following populations in both DTX-sensitive and DTX-resistant cells: E-cadherin positive and N-cadherin positive (Figure 6B, 6C), as well as CD44+ and CD44+/CD24- (Figure 7A, 7B). Consistent with their mesenchymal phenotype, DTX-resistant cells showed significantly reduced E-cadherin expression compared to DTX-sensitive cells, concomitant with an increase in N-cadherin expression, as determined by flow cytometry (Figure 6B, 6C) and immunoblotting (Figure 6D, left two panels). Notably, loss or downregulation of E-cadherin is associated with poor prognosis in PCa [16]. We also observed that loss of E-cadherin in the DTX-resistant cell lines was coupled with upregulation of Vimentin (Figure 6D, center panel) and transcription factors Snail and Twist (Figure 6D, right two panels). Vimentin, a well-established marker of EMT [30], was robustly and significantly upregulated in the PC3-DR cells compared to sensitive PC3 but its upregulation did not reach statistical significance in DU145-DR cells compared to sensitive DU145. Both Snail and Twist are known to repress E-cadherin expression, with Twist having a dual role in contributing to the upregulation of N-cadherin expression [15, 16, 30]. Taken together, these findings support growing evidence implicating EMT in PCa DTX-resistance [15, 16, 31].

In addition to these findings, we observed that the DTX-resistant cell populations displayed a higher frequency of cells expressing established CSC markers (Figure 7). CD44, one of these markers, is a multifunctional class I transmembrane glycoprotein that is highly expressed in most cancer types, where it contributes to tumor progression [32]. While we observed a significant proportion of PC3-DR cells with CD44+ expression compared to sensitive cells, there was no significant increase in the frequency of CD44+ cells in the DU145-DR population (Figure 7A, left two panels). However, because CD44 is expressed in almost all normal and cancer cells, specifically in normal prostate and PCa cells, there is a reported discrepancy and ambiguity regarding the functional aspects of this marker in prostate CSC maintenance [32]. This discrepancy is supported by our observation that sensitive DU145 cells showed high CD44+ expression (Figure 7A, 7B, left panels), and has been circumvented by using CD44 in combination with other markers to detect CSC subsets in PCa [32–36].

To better refine our detection of the putative CSC population in DTX-resistant cells, we used the well-validated combination of CD44+/CD24- [32, 35, 36]. CD24 is a luminal cell surface protein that contributes to metastasis and functions in cell-cell and cell-matrix interactions [32, 33, 36]. Because prostate CSCs arise from the basal cell compartment, the CD44+/CD24- marker combination is commonly used to identify these cells [32, 33]. We observed that both PC3-DR and DU145-DR cells contained substantial CD44+/CD24- subpopulations compared to the sensitive PC3 and DU145 cells (Figure 7A, 7B, right two panels).

Elevated aldehyde dehydrogenase (ALDH) activity is also emerging as a functional marker of a CSC-like phenotype because of its importance for CSC maintenance, signaling, and drug resistance [37]. To further confirm the acquisition of CSC-like characteristics in the DTX-resistant cells, we measured by flow cytometry the frequency of Aldefluor+ cells in our DTX-resistance cells compared to sensitive cells. Both PC3-DR and DU145-DR showed robust increase of ALDH activity compared to their sensitive counterparts (Figure 7C, 7D).

### Increased tumorsphere formation capacity and DTX-resistance in DU145-DR cells

**Figure 8:**
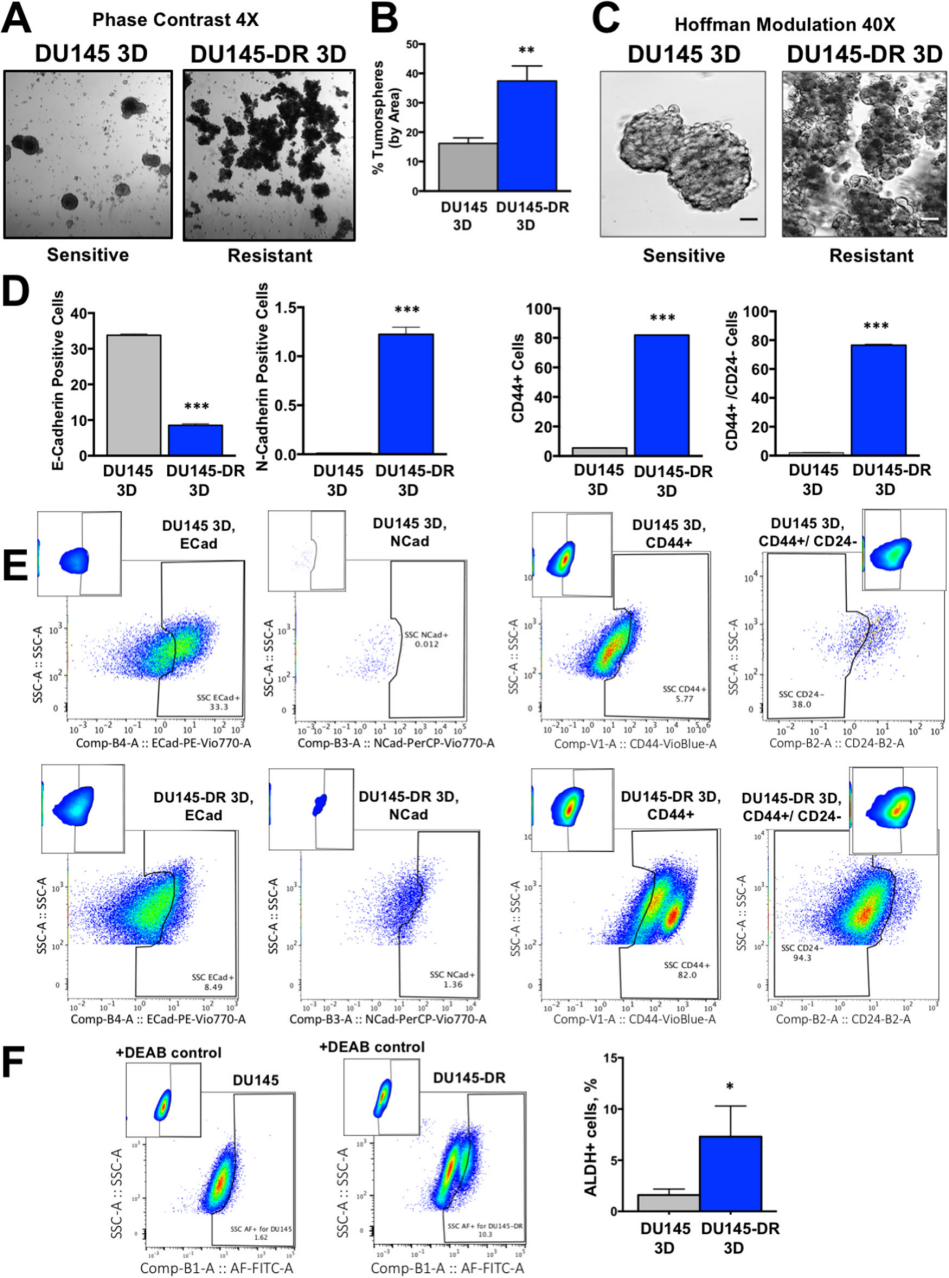
Tumorsphere formation capacity is higher in DTX-resistant DU145 cells compared to sensitive cells **(A)** Phase contrast microscopy images of DU145 and DU145-DR tumorspheres (3D) with **(B)** quantification of tumorsphere percentage using Image J software. **(C)** Tumorsphere morphology visualized using Hoffman modulation contrast microscopy (scale bar set at 40 μm). **(D)** Percent of live cells positive for the cell surface markers E-cadherin and N-cadherin, and CSC markers CD44+/CD24-, CD44+/CD24-, with **(E)** representative flow cytometry plots. **(F)** Representative flow cytometry plots showing increased ALDH activity (Aldefluor+) in DU145-DR tumorspheres as determined by aldefluor assay with bar graphs. All flow measurements were acquired from at least 3 independent experiments conducted separately. ^*^P< 0.05, ^**^P< 0.01, ^***^P< 0.001. Error bars represent mean ± SEM.

Upon confirmation of increased frequency of cells expressing EMT and CSC markers in the adherent (2D) DTX-resistant cell cultures, we sought to examine and compare tumorspheres (3D cultures) formed by sensitive and resistant DU145 cells. Tumorsphere formation is a widely used functional approach for enriching CSC populations, especially when specific surface CSC markers are not well defined or change with tumor heterogeneity [38–40]. We chose to focus these studies on the DU145 cell line because its DTX-sensitive cells formed large numbers of tumorspheres, consistent with previous reports that this cell line has a robust ability to form spheres even in the absence of external growth factors or drugs [34]. We observed that under tumorsphere-forming conditions, DU145-DR cells showed a 2.3-fold increase in tumorspheres compared to sensitive DU145 cells, as evidenced by phase contrast (4X) microscopic examination (Figure 8A, 8B). DU145-DR tumorspheres were loosely clustered, tethered together in grape-like clusters to form large aggregates (Figure 8C). This morphology was a stark difference from the tightly compact tumorspheres of sensitive DU145 cells.

Consistent with our analysis of adherent DTX-resistant cells (2D), flow cytometry analysis of DTX-resistant tumorspheres (3D) revealed a decreased frequency of E-cadherin expressing cells concomitant with increased frequency of N-cadherin expressing cells in the DU145-DR 3D cultures compared to DU145 3D cultures, (Figure 8D, 8E, left two panels). In addition, we detected increased frequencies of CD44+ and CD44+/CD24- populations in DU145-DR 3D compared to DU145 3D cultures (Figure 8D, 8E, right two panels). Furthermore, consistent with the 2D data, DU145-DR tumorspheres had a significantly higher number of Aldefluor+ cells than DU145 tumorspheres (Figure 8F).

**Figure 9:**
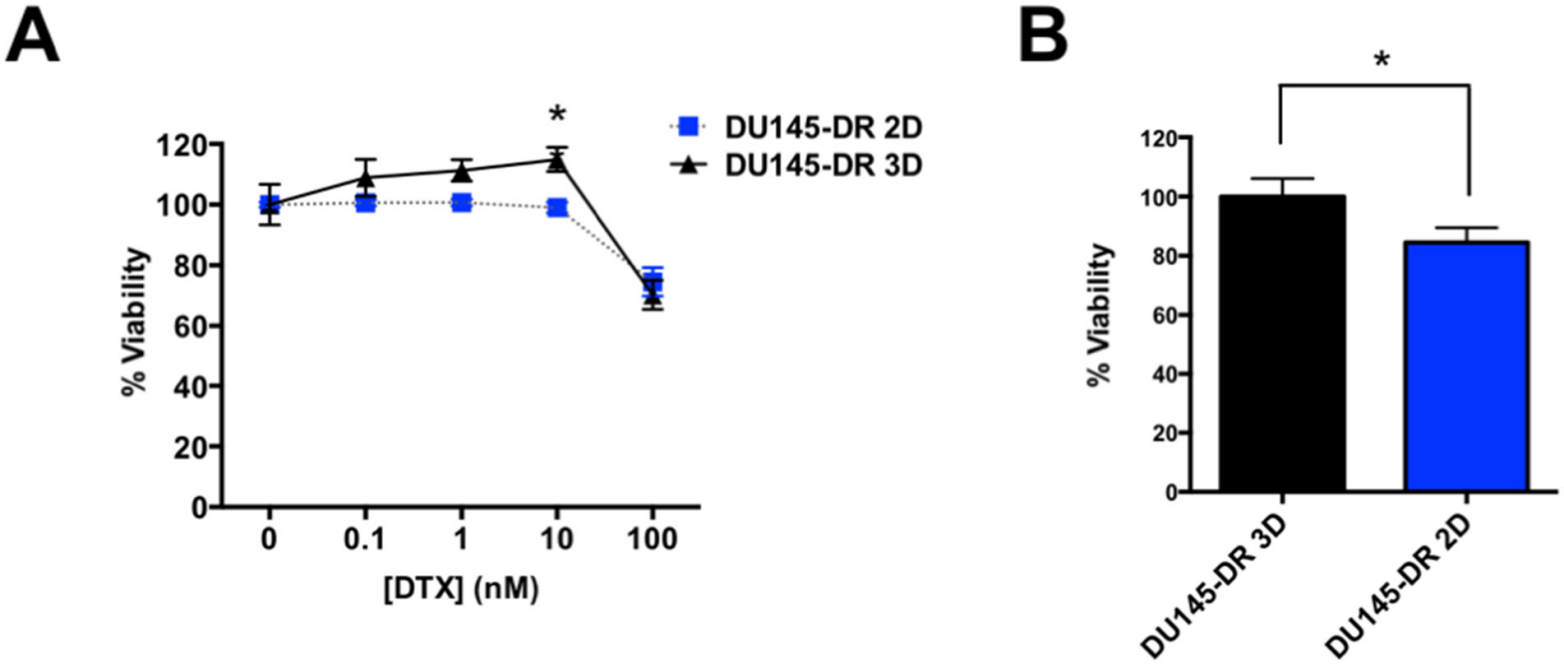
DU145-DR derived tumorspheres show increased resistance to DTX compared to DU145-DR adherent cells **(A)** DU145-DR adherent and tumorsphere cells treated with increasing concentrations of DTX (nM range) and % PI positive cells were measured via flow cytometry. **(B)** DU145-DR 3D tumorspheres were more resistant to 10 nM DTX than the adherent DU145-DR 2D cells as measured by % PI positive cells. All samples were normalized to untreated controls and to DU145-DR 3D percent viability. All measurements were acquired from at least 3 independent experiments with 3 biological replicates each. ^*^P< 0.05. Error bars represent mean ± SEM.

Other groups have demonstrated that tumorspheres derived from DU145 3D cells are more resistant to DTX treatment compared to DU145 2D cells [40, 41]. To further investigate the link between CSCs and DTX-resistance, we sought to determine if our DU145-DR tumorspheres were more resistant to DTX compared to DU145-DR 2D cells after exposure to increasing concentrations of DTX for 72 hours. Using propidium iodide (PI) staining of dead cells followed by flow cytometric analysis, we found that DU145-DR 3D tumorspheres were significantly more resistant to 10 nM DTX, the maintenance dose of DTX-resistant cell lines compared to the DU145-DR 2D cells grown in monolayer (Figure 9A, 9B). There were no statistical differences, however, at other lower or higher doses (Figure 9A), or at 24 or 48 hours time points (data not shown).

## DISCUSSION

There is a critical need for new drugs targeting non-traditional molecular targets that could be used alone or in combination with current agents for the treatment of therapy-resistant mCRPC. The present study used an RNA-seq approach to define transcriptomic signatures associated with DTX-resistance with the ultimate goal of identifying potentially novel therapeutic targets for overcoming this resistance. For these studies, we chose androgen-refractory PC3 and DU145 cells, which are widely used as cellular models that emulate late-stage mCRPC disease. While sensitive to DTX-treatment, these cell lines become resistant to the clinically relevant taxanes DTX, cabazitaxel, and paclitaxel upon incremental exposure to DTX and selection of surviving cells [8]. Resistance to both DTX and cabazitaxel is inevitable in mCRPC patients undergoing chemotherapy [2], but the mechanisms underlying this resistance remain to be clearly established.

PCa is fundamentally AR-driven especially in the context of disease initiation and progression. Because the intraprostatic response of PCa cells to androgens depends on the expression and sensitivity of AR, ADT has been a mainstay of PCa treatment and typically precedes taxane chemotherapy, although data from the recent “STAMPEDE” clinical trial showed improved patient survival when long-term primary ADT was combined with abiraterone acetate or DTX [42]. Constitutively active AR splice variants have been shown to be overexpressed in mCRPC and confer resistance to ADT by inhibiting the nuclear translocation of the androgen-AR complex [43–46]. A recent study suggested that AR splice variants may also affect sensitivity to taxanes and that tumors predominantly expressing the ARv7 variant, associated with ADT resistance, would also likely be resistant to DTX [47]. However, an independent group was unable to replicate these results under similar experimental conditions [48]. Furthermore, another group found that detection of ARv7 in circulating tumor cells of mCRPC patients was not associated with taxane-resistance and that certain patients with ARv7-positive status at baseline converted to ARv7-negative status during the course of taxane therapy, adding uncertainty to the clinical significance of this variant in patients receiving taxanes [49]. These discrepancies also contributed to our decision to focus on the AR-negative cell lines PC3 and DU145 for the present study.

Our RNA-seq analysis revealed over 1,200 genes that were differentially regulated in both the PC3-DR and DU145-DR cell lines. We focused on this set of overlap genes because differences in their expression are more likely to reflect transcriptomic changes induced by long-term DTX treatment regardless of the PCa cell type (e.g., PC3-bone metastasis vs. DU145-brain metastasis). Differentially expressed genes within this pool of overlap genes could potentially be exploited as therapeutic targets in heterogeneous metastatic prostate tumors that have acquired taxane resistance. GSEA of our RNA-seq data revealed several top ranked genes from the overlap dataset that an exhaustive PubMed literature review determined as being associated with tumor aggressiveness, chemoresistance, or CSC phenotype. Of note, GSEA yielded only one significant pathway enriched in the DTX-resistant cell lines compared to sensitive cells that yielded 8 genes positive for core enrichment. Of these, 4 genes (*ENPP1, CYC1, NDUFAB1*, and *MYC*) are associated with stem cell maintenance, acquisition or reprogramming [24–29], suggesting that in PC3 and DU145, DTX-resistance may be driven and maintained by the acquisition of CSC-like characteristics. Determining metabolic differences between DTX-sensitive and -resistant mCRPC cells will be imperative in future follow-up studies.

Using qPCR and immunoblotting, we validated several of the top upregulated genes in the DTX-resistant cells. These included genes associated with PCa aggressiveness, such as *FABP5* and *BOP1*, as well as genes implicated in CSC function such as *DPP4*, *TSPAN8*, *DNAJC12*, and *NES*. FABP5 is an intracellular lipid-binding protein that is emerging as a critical regulator of PCa cell proliferation and putative marker of aggressive PCa [50–53]. The robust FABP5 transcript and protein upregulation observed in the DTX-resistant cells suggest that this protein could be a promising target for the treatment of chemoresistant mCRPC. Another gene highly ranked in the GSEA was BOP1, an integral component of the ribosomal RNA processing machinery that contributes to colorectal tumorigenesis through promotion of cell migration and invasion [54, 55]. Interestingly, the *BOP1* gene is located in chromosome 8q24, a genomic region associated with PCa aggressiveness [54] that also encompasses *MYC* [56], one of the top upregulated genes in the DTX-resistant mCRPC cells revealed by our RNA-seq analysis.

An emerging stem cell marker, DPP4 (CD26) was also robustly upregulated in the DTX-resistant cells. DPP4 is a transmembrane glycoprotein that functions as an exopeptidase to promote cell migration through MMP-9, and contributes to the upregulation of CD44 [57]. This protein is upregulated in many cancers and associated with colon CSCs derived from DTX-resistant cells, which form larger and more tumorspheres [58–61]. The robust upregulation in protein expression observed in PC3-DR and DU145-DR suggest that DPP4 might be a prostate CSC marker that identifies a chemoresistant phenotype. The robust transcript and protein upregulation of TSPAN8 (TM4SF3) in the DTX-resistant cells also suggest a role for this protein in PCa chemoresistance. TSPAN8, promotes cell-to-cell communication by regulating integrins and other cell surface proteins [62], and its expression has been correlated with metastasis and worse prognosis in colon cancer where it contributes to cell motility through a complex with E-cadherin [63]. TSPAN8 is also considered a pancreatic CSC marker [64]. Genome splicing-sensitive microarray analysis revealed upregulation of TSPAN8 and DPP4 in DU145 tumorspheres compared to adherent DU145 2D cells [65].

DNAJC12, also known as Hsp40, has been implicated in cancer but its role in tumorigenesis is not clearly defined [66, 67]. The DNAJ family of proteins are considered regulators of CSC function [68], and DNAJC12 transcript expression was found to be upregulated in breast CSCs compared to adherent breast cancer cells [69]. NES, a cytoskeletal intermediate filament protein, has been associated with increased migration in PCa cells [70], and increased NES expression correlated with high tumor grade, invasive phenotype, and predictor of poor response to therapy [71]. Consistent with our observation that NES is robustly upregulated in DTX-resistant mCRPC cells with CSC-like characteristics, NES expression was previously reported in PCa tumorspheres that showed increased chemoresistance to paclitaxel [72], and was associated with a mesenchymal phenotype [73].

Oncomine data analysis comparing transcript expression of DPP4, TSPAN8, and NES between prostate tumor tissues and normal tissues revealed inconsistent upregulation of these genes in the different datasets. An explanation for this could be that the prostate tumors used to generate most of these gene expression datasets were not derived from advanced or chemoresistant disease. Alternatively, gene expression changes found in DTX-resistant cells occur in only a small subset of cells, most likely those with stemness properties. Since tumors contain varying proportions of cells with and without stemness properties, it will be difficult to consistently detect global gene expression changes in CSCs present in PCa tissues since they comprise a minority of the population. A limitation of the Oncomine database is the assessment of gene expression in normal vs. PCa tissues without extensive clinical data (type of treatment, tumor stage, etc.) for several of the datasets. Therefore, to further validate the expression of selected genes of interest in clinically relevant tissues, it will be important in future studies to obtain mCRPC biospecimens with annotated clinical data from patients with and without taxane treatment, and that responded to or failed the treatment. We recognize, however, the intrinsic difficulties in obtaining such biospecimens.

The beneficial effects of chemotherapeutic drugs like DTX are hindered by the development of chemoresistance. Emerging evidence demonstrates that a small population of CSCs present within the tumors possesses multiple redundant mechanisms that facilitate tumor cell survival in the presence of therapeutic agents [12, 14, 17]. In addition, the relatively non-proliferative state of CSCs makes this small population of cells intrinsically resistant to conventional chemotherapies, most of which target rapidly dividing cells. This resistant population comprises a tumor cell reserve that persists even after anti-proliferative treatments and repopulates the tumor in metastatic sites [12]. The emerging role of CSCs in the acquisition of PCa chemoresistance [12, 15, 17, 74], and the observation that highly-ranked genes in our RNA-seq analysis were associated with a CSC-like phenotype or genetic program, led us to characterize this population in DTX-sensitive and -resistant mCRPC PC3 and DU145 cells. Putative CSCs are typically identified based on the presence and/or absence of several cell surface markers, the combination of which is specific for the CSC-like phenotype identified in a particular tumor type [71]. Our observation that the PC3-DR and DU145-DR cell cultures were enriched with cell populations expressing several of these CSC markers, including significantly elevated ALDH activity compared to DTX-sensitive parental cells, is consistent with the acquisition of CSC-like characteristics. Furthermore, our finding that DU145-DR cells have an enhanced capacity to form tumorspheres (3D) and increased ALDH activity compared to DU145 tumorspheres, is an indicator of the increased CSC-like characteristics of the resistance cells. In addition, our DU145-DR tumorspheres showed increased resistance to 10 nM DTX, a clinically relevant dose, compared to adherent DU145-DR cells (2D), suggesting that a CSC-like phenotype contributes to enhanced DTX resistance. An accurate assessment of the increased tumorigenic potential of DTX-resistant cells with CSC-like characteristics would be more effectively achieved through *in vivo* studies with animal models using enriched CSC populations acquired by cell sorting.

Targeting CSCs is a promising approach to circumvent tumor chemoresistance [14, 17]. Current strategies focus on targeting signaling pathways upregulated in stem cells that are specific to their function, including the Hedgehog, Wnt, Notch, and NFkB pathways [12]. The present RNA-seq study provides additional candidate genes and molecular pathways for potential therapeutic targeting, and contributes to the emerging body of evidence linking CSCs to PCa chemoresistance. Future pre-clinical studies will focus on establishing mechanistic roles of specific genes identified in our RNA-seq analysis in the maintenance of prostate CSCs and driving taxane resistance, validating their expression in clinical biospecimens derived from PCa patients that failed taxane therapy, and investigating their potential as therapeutic targets. It will also be important to further define PCa cell-type dependent differences in the expression of CSC and chemoresistance-associated genes, as our RNA-seq analysis demonstrated that PC3-DR and DU145-DR cells have differentially regulated genes that are unique to each of these cell lines. This would be critical for tackling the high heterogeneity that characterizes prostate tumors.

## MATERIALS AND METHODS

### Cell lines and culture

The metastatic PCa cell lines PC3 and DU145 were purchased from the American Type Culture Collection (ATCC, Cat# ATCC-CRL-1435 and ATCC-HTB-81, respectively). Cells were cultured as recommended by the supplier in RPMI medium (Thermo Fisher Scientific) supplemented with 10% fetal bovine serum, penicillin/streptomycin, and gentamicin. Cells were maintained in a humidified incubator with 5% CO_2_ at 37°C. DTX-resistant (DR) PC3 and DU145 were developed as described previously [18]. Briefly, PC3 and DU145 cells were cultured in media containing 1 nM DTX (LC Laboratories Cat# D-1000) and surviving cells were passaged four times before increasing the concentration of DTX. This was repeated until resistant cells could be maintained with minimal cell death in the presence of 11 nM DTX.

Short tandem repeat (STR) profiling is a recommended and validated method for authentication of human cell lines and tissues [75]. The importance of cell line authentication is highlighted by the NIH initiative for rigor and reproducibility in scientific research [76], and is particularly important for scientific studies such as the present one that use established cancer cell lines for pre-clinical mechanistic studies. We utilized the STR service provided by ATCC (Cat# ATCC 135-XV) to authenticate the PC3 and DU145 cell lines used in this study. Both cell lines matched their respective database profiles. The DTX-resistant PC3-DR and DU145-DR cell lines were derived from these validated PC3 and DU145 parental cell lines.

### RNA isolation and RNA-seq library preparation and sequencing

Total RNA was extracted from DTX-sensitive and -resistant PC3 and DU145 cells using the miRNeasy Mini Kit (Qiagen Cat# 217004). RNA-seq library construction and sequencing was performed at the Loma Linda University School of Medicine Center for Genomics. RNA-seq library was constructed using the TruSeq Stranded mRNA Low Sample Preparation protocol (Illumina; Cat# RS-1229004DOC). Two μg of total RNA were used as input. Each RNA sample was spiked with 1:100 ERCC RNA spike-in control mix 1 (Life Technologies, Cat# 4456740) prior to the first step of the protocol. All the recommended controls were used during subsequent steps including an End Repair Control, A-Tailing Control, and Ligation Control. The RNA-seq libraries were quantified using Qubit 3.0, and the quality of RNA-seq libraries was checked on Agilent TapeStation. All RNA-seq libraries were sequenced on Illumina HiSeq 4000 at the Loma Linda University Center for Genomics, with 150 bpx2, Paired-End. Quality control was confirmed (see Supplementary Figures 3 and 4).

### RNA-seq data analysis

For mRNA-seq data visualization and analysis, we utilized pipelines that integrated the QC (FastQC, ShortRead), trimming process (trimmomatic), alignment (Tophat2), reads quantification (cufflinks), and differentially expressed gene (DEG) analysis (cuffdiff) as described previously [77]. Briefly, the RNA-seq raw fastq data were first trimmed using Trimmomatic (V0.35). The trimmed reads were aligned to the human reference genome (NCBI GRCh38) with TopHat V2.1.1 with default parameter settings. The aligned bam files were then processed using Cufflinks V2.2.1 for gene quantification. Reads were then mapped to ERCC transcripts and quantified using TopHat V2.1.1 and Cufflinks V2.1.1 with default parameter settings. Genes with FPKM ≥ 1 in all samples were used for DEG analysis. Differentially expressed genes (DEGs) were identified by Cuffdiff with FDR > 0.05, and fold change (FC) > 2.

Hierarchical clustering heat map and PCA of global genes for all cell lines were performed with “R” program (http://cran.r-project.org/) [78] and Partek Genomics Suite 6.6, respectively. GSEA (v3.0, Broad Institute) [79, 80], was performed to compare parental DTX-sensitive PC3 and DU145 with DTX-resistant DU145-DR and PC3-DR. Gene sets were obtained from published gene signatures in the Molecular Signatures Database v1.0 (MSigDB). Analysis was run with 1,000 permutations and a classic statistic. Normalized enrichment score and *p*-values were measured to find enrichments with statistical significance (*p*<0.05).

### Quantitative reverse transcription PCR (RT-qPCR)

For confirmation of RNA-seq results, we selected specific genes for independent in-house validation of their differential regulation in DTX-sensitive vs. -resistant cell lines. Briefly, total RNA was extracted from cell lines using the RNAprotect reagent (Qiagen Cat# 76526) and the RNeasy plus mini kit (Qiagen Cat# 74134). RNA (0.5 μg) was reverse transcribed into cDNA using iScript cDNA synthesis kit (Bio-Rad Cat# 1708891). Primer sequences for gene validation were commercially synthesized by Integrative DNA Technologies (IDT) (see Supplementary Table 2). Quantitative polymerase chain reaction (qPCR) was performed on the MyiQ real-time PCR and CFX96 Touch Real-Time PCR (Bio-Rad) detection system using iQSYBR Green Supermix (Bio-Rad Cat # 170-8882) according to the manufacturer's instructions. The cycling conditions were 95°C for 15 min, 95°C for 15s, 60°C for 60s for 35 cycles, followed by melt analysis from 60 to 95°C. Expression levels were normalized to the housekeeping gene glyceraldehyde 3-phosphate dehydrogenase (GAPDH). Samples were analyzed in at least four independent biological replicates performed experimentally in triplicates.

### Immunoblotting procedures

Whole cell lysates were prepared and the protein concentration in the lysates was determined using the BioRad DC Protein Assay Kit (Cat# 5000112) to ensure equal loading of proteins per lane. Bands were separated by SDS-PAGE (NuPAGE 4-12%, Thermo-Fisher Scientific) followed by transfer to polyvinyl difluoride membrane (Millipore). Membranes were blocked with either 5% dry milk solution or 5% bovine serum albumin both prepared in TBS-T buffer (20 mM Tris-HCL, pH 7.6, 140 mM NaCl, 0.2% Tween 20) and probed with the following primary antibodies: Rabbit anti-LEDGF/p75 (1:1000, Bethyl Laboratories Cat # A300-848A), mouse anti-clusterin alpha chain (1:1000, Millipore Cat# 05-354), rabbit anti-MDR1/ABCB1 (1:1000 Cell Signaling Cat# 13342), rabbit anti-DPP4/CD26 (1:3000, Millipore Cat# MABF752), mouse anti-Nestin (1:1000 Millipore Cat# MAB5326), rabbit anti-DNAJC12 (1:500, Novus Cat# NBP1-57718), rabbit anti-FABP5 (1:5000; a kind gift from Marino De Leon, Loma Linda University, Loma Linda, CA), mouse anti-Snail (1:1000, Cell Signaling Cat# 3895S), mouse anti-Twist (1:200, Santa Cruz Cat# sc-81417), rat anti-Vimentin (1:8000, R&D Systems Cat# MAB2105-SP), rabbit anti-TGM2 (1:1000, Cell Signaling Cat# 3557), rabbit anti-BOP1 (1:1000, Bethyl Cat# A302-148A-M-1), mouse anti-E-cadherin (1:500, BD Biosciences Cat# 610182), or mouse anti-N-cadherin (1:200, Abcam Cat# ab12221). The mouse anti-TSPAN8 primary antibody was from Celine Greco and Claude Boucheix (1:2000) [81].

Following several washes with TBS-T, membranes were incubated with the appropriate horseradish peroxidase (HRP)-conjugated secondary antibodies (anti-mouse IgG and anti-rabbit IgG, Cell Signaling Cat # 7076 and 7074, respectively; goat anti rat, Santa Cruz Cat# sc-2032). HRP-β-actin was utilized as a loading control (Cell Signaling Cat # 5125). After 2-hour incubation with secondary antibodies, the membranes were washed several times with TBS-T, and protein bands were detected by enhanced chemiluminescence (Thermo Fisher Scientific, Cat# 34580). Bands were quantified using Image J software (National Institutes of Health) and normalized to β-actin control. Samples were analyzed in at least 3 independent experiments using at least 3 biological replicates.

### Bioinformatics analysis of oncomine cancer gene microarray database

For analysis of mRNA expression of genes of interest in PCa and normal prostate tissues, we selected 16 datasets from the Oncomine database (Compendia Biosciences; Ann Arbor, MI; www.oncomine.org). These datasets, derived from gene microarray analyses of PCa and normal prostate tissues, provide fold-change data for gene expression with *P* values calculated by Oncomine using Student's *t*-tests. The Grasso dataset included 35 castration-resistant metastatic PCa, 59 localized PCa, and 28 benign prostate tissue specimens while the Varambally dataset included 6 hormone-refractory metastatic PCa samples in addition to 7 localized PCa, and 6 normal prostate samples. This allowed us to compare the transcript expression between these 3 categories of tissues in our genes of interest.

### Tumorsphere forming assays

Cells were cultured in 6-well non-tissue culture treated plates at a density of 25,000 cells/ml, and suspended in F12K/RPMI supplemented with 1% knockout serum replacement (Fisher Scientific Cat# 10828028), 20 ng/ml human EGF (Millipore Sigma Cat# E9644), 10 ng/ml human bFGF (PeproTech Cat# 100-18B), 0.1% of albumin solution 35% in PBS (Sigma Cat# 091M8416), 1% Pen-Strep, 0.1% insulin (Millipore Sigma Cat# 10516), and 0.1% selenium (Millipore Sigma Cat# 229865). After 24 hours the floating cells were collected and cultured in separate plates in the medium described above. Cells were left for 14 days adding or replacing medium as necessary to maintain growth. Images of cells were taken after at least 14 days post-plating using an Olympus IX70 microscope with phase contrast and Hoffman modulation contrast and equipped with a SPOT RT3 imaging system. Using phase contrast 4X images and Image J software, tumorsphere formation was quantified as percent area in at least four independent experiments.

### Flow cytometric analysis of stem cell markers, ALDH activity, and cell death

Adherent PC3-DR and DU145-DR cells were cultured in monolayer to 80-90% confluency prior to collection for multicolor flow cytometric analysis of putative CSC markers. Cells were washed with PBS and harvested using a solution containing 0.25% Trypsin and 2.21mM EDTA (Corning Cat# 25-053-Cl), followed by incubation in fresh fully supplemented RPMI medium containing 10% FBS for 30 minutes to allow for N-Cadherin and E-Cadherin recycling following enzymatic cleavage. In parallel, tumorspheres derived from PC3-DR or DU145-DR cells were collected and dissociated using 0.25% Trypsin/2.21mM EDTA solution, followed by neutralization with fresh fully-supplemented medium. Following the 30-minute recovery period, cells were then labeled with antibodies against CD44, CD24, N-Cadherin, E-Cadherin, or annexin-V for 15 minutes at room temperature (see Supplementary Table 3 for antibody specifications). Cells were washed and resuspended in annexin-V binding buffer (50 mM HEPES, 700 mM NaCl, 12.5 mM CaCl_2_; pH 7.4) and analyzed immediately on a MACSQuant Analyzer 10 equipped with violet, blue, and red lasers (Miltenyi Biotec). Post-acquisition data analysis was performed using FlowJo version 10.08.1 (BD).

ALDH activity was detected using Aldefluor assay kit purchased from Stem Cell Technologies (Cat# 01700) and performed according to manufacturer's instructions. Briefly, 2D and 3D cells were prepared and harvested as described above. 400,000 cells were resuspended in 200 μl of aldefluor buffer and 2 μl of aldefluor reagent to form the “test” sample. 200 μl of that text mix were then immediately transferred to another microcentrifuge tube containing 2 μl of DEAB reagent to inactivate the aldefluor reagent and become the “control” sample. Both the control and test sample were incubated for 45 minutes at 37°C. Samples were then centrifuged and resuspended in aldefluor buffer to be analyzed immediately on the MACSQuant Analyzer. Post-acquisition data analysis was performed using FlowJo version 10.08.1 (BD) with gates being drawn on the control DEAB+ samples for each cell line 2D and 3D.

Initial gates for intact cells using FSC-A/SSC-A light scatter and doublet discrimination using FSC-H/FSC-A profiles. Single-stained samples were used to define compensation matrices. Following compensation, dead cells were excluded based on annexin-V positivity and only live cells were assessed for putative CSC marker expression. Gate placements were defined using Fluorescence-Minus-One (FMO) controls using SSC-A versus marker of interest (see Supplementary Figure 5 for gating strategy and Supplementary Table 4 for staining strategy for FMO detection). Data are presented as percent of live cells staining positive for each designated marker and are representative of at least 3 independent experiments.

### Detection of cell viability by propidium iodide staining

PC3 and DU145 cells, DTX-sensitive or -resistant, were seeded in 6-well cluster plates at 1.25×10^5^ cells per well and allowed to adhere for 24 hours. Separately, PC3-DR and DU145-DR cells were seeded in non-adherent conditions in 6-well cluster plates at 5 x10^4^ cells per well. Cells were then treated with increasing concentrations of DTX (0.1, 1, 10, 100, 1000 nM) for 72 hours, followed by PI staining using the Dead Cell Apoptosis Kit for flow cytometry (Life Technologies, Cat# V13242) according to the manufacturer's instructions. Briefly, adherent cells were detached from culture using 0.25% Trypsin/2.21mM EDTA solution for 30 seconds, followed by neutralization using complete RPMI medium containing 10% FBS. Cells grown in non-adherent conditions were collected from culture medium and dissociated using 0.25% Trypsin/2.21mM EDTA solution for 30 seconds, followed by neutralization using complete medium containing 10% FBS. Cells were washed with PBS, suspended in annexin-V binding buffer and stained with PI (1μg/mL final concentration) for 15 minutes at room temperature in the dark, then immediately analyzed on a MACSQuant Analyzer. Following exclusion of debris and doublet events, single-stained samples were used to define compensation matrices and experimental gates. Data are presented as percentage of cells staining negative for PI (percent viability) (see Supplementary Figure 6 for gating strategy).

### Statistical analysis

Statistical analysis and graph generation was performed using GraphPad Prism version 6.0c for Mac OSX (GraphPad Software, La Jolla, California USA, www.graphpad.com). Fold change differences in both qPCR, immunoblotting, Oncomine data, and tumorsphere percent area were analyzed using Student's *t*-test. Results were considered significant at *P* < 0.05. One-Way ANOVA was used for the analysis of results from PI staining experiments comparing percent viability in the resistant 2D (adherent) compared to resistant 3D (tumorspheres) cultures.

## SUPPLEMENTARY MATERIALS FIGURES AND TABLES



## Abbreviations

mCRPC: metastatic castration-resistant prostate cancer
DTX: docetaxel
RNA-seq: RNA sequencing
GSEA: Gene Set Enrichment Analysis
CSC: cancer stem cell
PCa: prostate cancer
PSA: prostate-specific antigen
CLU: clusterin
EMT: epithelial-to-mesenchymal transition
NGS: Next Generation Sequencing
LEDGF/p75: Lens Epithelium-Derived Growth Factor
AR: Androgen Receptor
ABCB1: ATP-binding cassette sub-family B member 1
ALDH: aldehyde dehydrogenase
3D-PCA: Principal Component Analysis
ENPP1: ectonucleotide phosphodiesterase 1
CYC1: cytochrome c-1
NDUFAB1: NADH dehydrogenase 1 alpha/beta subcomplex 1
MYC: v-myc myelocytomatosis viral oncogene homolog
DPP4: dipeptidyl peptidase 4
TSPAN8: tetraspanin 8
NES: nestin
DNAJC12: DNAJ heat shock protein family member C12
FABP5: fatty acid binding protein 5
BOP1: block of proliferation 1
TGM2: transglutaminase 2
ABCC3: ATP binding cassette subfamily C member 3
ATCC: American Type Culture Collection
STR: short tandem repeat
DEG: differentially expressed gene
IDT: Integrative DNA technologies
qPCR: quantitative polymerase chain reaction
GAPDH: glyceraldehyde 3-phosphate dehydrogenase
HRP: horseradish peroxidase
FMO: fluorescence-minus-one
DR: docetaxel resistant
PI: propidium iodide.

## References

[1] Siegel RL, Miller KD, Jemal A (2018). Cancer statistics, 2018. CA Cancer J Clin.

[2] Chandrasekar T, Yang JC, Gao AC, Evans CP (2015). Targeting molecular resistance in castration-resistant prostate cancer. BMC Med.

[3] Feldman BJ, Feldman D (2001). The development of androgen-independent prostate cancer. Nat Rev Cancer.

[4] Colloca G, Venturino A, Checcaglini F (2012). Second-line chemotherapy in metastatic docetaxel-resistant prostate cancer: a review. Med Oncol.

[5] Tannock IF, de Wit R, Berry WR, Horti J, Pluzanska A, Chi KN, Oudard S, Theodore C, James ND, Turesson I, Rosenthal MA, Eisenberger MA (2004). Docetaxel plus prednisone or mitoxantrone plus prednisone for advanced prostate cancer. N Engl J Med.

[6] Beltran H, Beer TM, Carducci MA, de Bono J, Gleave M, Hussain M, Kelly WK, Saad F, Sternberg C, Tagawa ST, Tannock IF (2011). New therapies for castration-resistant prostate cancer: efficacy and safety. Eur Urol.

[7] Gillessen S, Omlin A, Attard G, de Bono JS, Efstathiou E, Fizazi K, Halabi S, Nelson PS, Sartor O, Smith MR, Soule HR, Akaza H, Beer TM (2015). Management of patients with advanced prostate cancer: recommendations of the St Gallen Advanced Prostate Cancer Consensus Conference (APCCC) 2015. Ann Oncol.

[8] Rios-Colon L, Cajigas-Du Ross CK, Basu A, Elix C, Alicea-Polanco I, Sanchez TW, Radhakrishnan V, Chen CS, Casiano CA (2017). Targeting the stress oncoprotein LEDGF/p75 to sensitize chemoresistant prostate cancer cells to taxanes. Oncotarget.

[9] Djeu JY, Wei S (2009). Clusterin and chemoresistance. Adv Cancer Res.

[10] Chi KN, Higano CS, Blumenstein B, Ferrero JM, Reeves J, Feyerabend S, Gravis G, Merseburger AS, Stenzl A, Bergman AM, Mukherjee SD, Zalewski P, Saad F (2017). Custirsen in combination with docetaxel and prednisone for patients with metastatic castration-resistant prostate cancer (SYNERGY trial): a phase 3, multicentre, open-label, randomised trial. Lancet Oncol.

[11] Beer TM, Hotte SJ, Saad F, Alekseev B, Matveev V, Flechon A, Gravis G, Joly F, Chi KN, Malik Z, Blumenstein B, Stewart PS, Jacobs CA (2017). Custirsen (OGX-011) combined with cabazitaxel and prednisone versus cabazitaxel and prednisone alone in patients with metastatic castration-resistant prostate cancer previously treated with docetaxel (AFFINITY): a randomised, open-label, international, phase 3 trial. Lancet Oncol.

[12] Leao R, Domingos C, Figueiredo A, Hamilton R, Tabori U, Castelo-Branco P (2017). Cancer Stem Cells in Prostate Cancer: Implications for Targeted Therapy. Urol Int.

[13] Shen MM, Abate-Shen C (2010). Molecular genetics of prostate cancer: new prospects for old challenges. Genes Dev.

[14] Ni J, Cozzi P, Hao J, Duan W, Graham P, Kearsley J, Li Y (2014). Cancer stem cells in prostate cancer chemoresistance. Curr Cancer Drug Targets.

[15] Marin-Aguilera M, Codony-Servat J, Reig O, Lozano JJ, Fernandez PL, Pereira MV, Jimenez N, Donovan M, Puig P, Mengual L, Bermudo R, Font A, Gallardo E (2014). Epithelial-to-mesenchymal transition mediates docetaxel resistance and high risk of relapse in prostate cancer. Mol Cancer Ther.

[16] Hanrahan K, O'Neill A, Prencipe M, Bugler J, Murphy L, Fabre A, Puhr M, Culig Z, Murphy K, Watson RW (2017). The role of epithelial-mesenchymal transition drivers ZEB1 and ZEB2 in mediating docetaxel-resistant prostate cancer. Mol Oncol.

[17] Yun EJ, Zhou J, Lin CJ, Hernandez E, Fazli L, Gleave M, Hsieh JT (2016). Targeting Cancer Stem Cells in Castration-Resistant Prostate Cancer. Clin Cancer Res.

[18] Basu A, Cajigas-Du Ross CK, Rios-Colon L, Mediavilla-Varela M, Daniels-Wells TR, Leoh LS, Rojas H, Banerjee H, Martinez SR, Acevedo-Martinez S, Casiano CA (2016). LEDGF/p75 Overexpression Attenuates Oxidative Stress-Induced Necrosis and Upregulates the Oxidoreductase ERP57/PDIA3/GRP58 in Prostate Cancer. PLoS One.

[19] Koltai T (2014). Clusterin: a key player in cancer chemoresistance and its inhibition. Onco Targets Ther.

[20] Zhong B, Sallman DA, Gilvary DL, Pernazza D, Sahakian E, Fritz D, Cheng JQ, Trougakos I, Wei S, Djeu JY (2010). Induction of clusterin by AKT--role in cytoprotection against docetaxel in prostate tumor cells. Mol Cancer Ther.

[21] O'Neill AJ, Prencipe M, Dowling C, Fan Y, Mulrane L, Gallagher WM, O'Connor D, O'Connor R, Devery A, Corcoran C, Rani S, O'Driscoll L, Fitzpatrick JM (2011). Characterisation and manipulation of docetaxel resistant prostate cancer cell lines. Mol Cancer.

[22] Gravina GL, Mancini A, Colapietro A, Marampon F, Sferra R, Pompili S, Biordi LA, Iorio R, Flati V, Argueta C, Landesman Y, Kauffman M, Shacham S (2017). Pharmacological treatment with inhibitors of nuclear export enhances the antitumor activity of docetaxel in human prostate cancer. Oncotarget.

[23] Hwang C (2012). Overcoming docetaxel resistance in prostate cancer: a perspective review. Ther Adv Med Oncol.

[24] Bageritz J, Goidts V (2014). Functional characterization of ENPP1 reveals a link between cell cycle progression and stem-like phenotype in glioblastoma. Mol Cell Oncol.

[25] Takahashi RU, Miyazaki H, Takeshita F, Yamamoto Y, Minoura K, Ono M, Kodaira M, Tamura K, Mori M, Ochiya T (2015). Loss of microRNA-27b contributes to breast cancer stem cell generation by activating ENPP1. Nat Commun.

[26] Rossello RA, Pfenning A, Howard JT, Hochgeschwender U (2016). Characterization and genetic manipulation of primed stem cells into a functional naive state with ESRRB. World J Stem Cells.

[27] Kopp S, Sahana J, Islam T, Petersen AG, Bauer J, Corydon TJ, Schulz H, Saar K, Huebner N, Slumstrup L, Riwaldt S, Wehland M, Infanger M (2018). The role of NFkappaB in spheroid formation of human breast cancer cells cultured on the Random Positioning Machine. Sci Rep.

[28] Poli V, Fagnocchi L, Fasciani A, Cherubini A, Mazzoleni S, Ferrillo S, Miluzio A, Gaudioso G, Vaira V, Turdo A, Giaggianesi M, Chinnici A, Lipari E (2018). MYC-driven epigenetic reprogramming favors the onset of tumorigenesis by inducing a stem cell-like state. Nat Commun.

[29] Dang CV, Le A, Gao P (2009). MYC-induced cancer cell energy metabolism and therapeutic opportunities. Clin Cancer Res.

[30] Garg M (2013). Epithelial-mesenchymal transition - activating transcription factors - multifunctional regulators in cancer. World J Stem Cells.

[31] Puhr M, Hoefer J, Schafer G, Erb HH, Oh SJ, Klocker H, Heidegger I, Neuwirt H, Culig Z (2012). Epithelial-to-mesenchymal transition leads to docetaxel resistance in prostate cancer and is mediated by reduced expression of miR-200c and miR-205. Am J Pathol.

[32] Jaggupilli A, Elkord E (2012). Significance of CD44 and CD24 as cancer stem cell markers: an enduring ambiguity. Clin Dev Immunol.

[33] Rybak AP, Bristow RG, Kapoor A (2015). Prostate cancer stem cells: deciphering the origins and pathways involved in prostate tumorigenesis and aggression. Oncotarget.

[34] Rybak AP, He L, Kapoor A, Cutz JC, Tang D (2011). Characterization of sphere-propagating cells with stem-like properties from DU145 prostate cancer cells. Biochim Biophys Acta.

[35] Harris KS, Kerr BA (2017). Prostate Cancer Stem Cell Markers Drive Progression, Therapeutic Resistance, and Bone Metastasis. Stem Cells Int.

[36] Sharpe B, Beresford M, Bowen R, Mitchard J, Chalmers AD (2013). Searching for prostate cancer stem cells: markers and methods. Stem Cell Rev.

[37] Clark DW, Palle K (2016). Aldehyde dehydrogenases in cancer stem cells: potential as therapeutic targets. Ann Transl Med.

[38] Rappa G, Mercapide J, Anzanello F, Prasmickaite L, Xi Y, Ju J, Fodstad O, Lorico A (2008). Growth of cancer cell lines under stem cell-like conditions has the potential to unveil therapeutic targets. Exp Cell Res.

[39] Ponti D, Costa A, Zaffaroni N, Pratesi G, Petrangolini G, Coradini D, Pilotti S, Pierotti MA, Daidone MG (2005). Isolation and in vitro propagation of tumorigenic breast cancer cells with stem/progenitor cell properties. Cancer Res.

[40] Zhang L, Jiao M, Li L, Wu D, Wu K, Li X, Zhu G, Dang Q, Wang X, Hsieh JT, He D (2012). Tumorspheres derived from prostate cancer cells possess chemoresistant and cancer stem cell properties. J Cancer Res Clin Oncol.

[41] Portillo-Lara R, Alvarez MM (2015). Enrichment of the Cancer Stem Phenotype in Sphere Cultures of Prostate Cancer Cell Lines Occurs through Activation of Developmental Pathways Mediated by the Transcriptional Regulator DeltaNp63alpha. PLoS One.

[42] Sydes MR, Spears MR, Mason MD, Clarke NW, Dearnaley DP, de Bono JS, Attard G, Chowdhury S, Cross W, Gillessen S, Malik ZI, Jones R, Parker CC (2018). Adding abiraterone or docetaxel to long-term hormone therapy for prostate cancer: directly randomised data from the STAMPEDE multi-arm, multi-stage platform protocol. Ann Oncol.

[43] Dehm SM, Schmidt LJ, Heemers HV, Vessella RL, Tindall DJ (2008). Splicing of a novel androgen receptor exon generates a constitutively active androgen receptor that mediates prostate cancer therapy resistance. Cancer Res.

[44] Guo Z, Yang X, Sun F, Jiang R, Linn DE, Chen H, Chen H, Kong X, Melamed J, Tepper CG, Kung HJ, Brodie AM, Edwards J (2009). A novel androgen receptor splice variant is up-regulated during prostate cancer progression and promotes androgen depletion-resistant growth. Cancer Res.

[45] Hu R, Dunn TA, Wei S, Isharwal S, Veltri RW, Humphreys E, Han M, Partin AW, Vessella RL, Isaacs WB, Bova GS, Luo J (2009). Ligand-independent androgen receptor variants derived from splicing of cryptic exons signify hormone-refractory prostate cancer. Cancer Res.

[46] Sun S, Sprenger CC, Vessella RL, Haugk K, Soriano K, Mostaghel EA, Page ST, Coleman IM, Nguyen HM, Sun H, Nelson PS, Plymate SR (2010). Castration resistance in human prostate cancer is conferred by a frequently occurring androgen receptor splice variant. J Clin Invest.

[47] Thadani-Mulero M, Portella L, Sun S, Sung M, Matov A, Vessella RL, Corey E, Nanus DM, Plymate SR, Giannakakou P (2014). Androgen receptor splice variants determine taxane sensitivity in prostate cancer. Cancer Res.

[48] Shan X, Danet-Desnoyers G, Aird F, Kandela I, Tsui R, Perfito N, Iorns E (2018). Replication study: androgen receptor splice variants determine taxane sensitivity in prostate cancer. PeerJ.

[49] Antonarakis ES (2015). Predicting treatment response in castration-resistant prostate cancer: could androgen receptor variant-7 hold the key?. Expert Rev Anticancer Ther.

[50] Kawaguchi K, Kinameri A, Suzuki S, Senga S, Ke Y, Fujii H (2016). The cancer-promoting gene fatty acid-binding protein 5 (FABP5) is epigenetically regulated during human prostate carcinogenesis. Biochem J.

[51] Bao Z, Malki MI, Forootan SS, Adamson J, Forootan FS, Chen D, Foster CS, Rudland PS, Ke Y (2013). A novel cutaneous Fatty Acid-binding protein-related signaling pathway leading to malignant progression in prostate cancer cells. Genes Cancer.

[52] Fujita K, Kume H, Matsuzaki K, Kawashima A, Ujike T, Nagahara A, Uemura M, Miyagawa Y, Tomonaga T, Nonomura N (2017). Proteomic analysis of urinary extracellular vesicles from high Gleason score prostate cancer. Sci Rep.

[53] Al-Jameel W, Gou X, Forootan SS, Al Fayi MS, Rudland PS, Forootan FS, Zhang J, Cornford PA, Hussain SA, Ke Y (2017). Inhibitor SBFI26 suppresses the malignant progression of castration-resistant PC3-M cells by competitively binding to oncogenic FABP5. Oncotarget.

[54] Chung KY, Cheng IK, Ching AK, Chu JH, Lai PB, Wong N (2011). Block of proliferation 1 (BOP1) plays an oncogenic role in hepatocellular carcinoma by promoting epithelial-to-mesenchymal transition. Hepatology.

[55] Killian A, Sarafan-Vasseur N, Sesboue R, Le Pessot F, Blanchard F, Lamy A, Laurent M, Flaman JM, Frebourg T (2006). Contribution of the BOP1 gene, located on 8q24, to colorectal tumorigenesis. Genes Chromosomes Cancer.

[56] Nowinski S, Santaolalla A, O'Leary B, Loda M, Mirchandani A, Emberton M, Van Hemelrijck M, Grigoriadis A (2018). Systematic identification of functionally relevant risk alleles to stratify aggressive versus indolent prostate cancer. Oncotarget.

[57] Yu DM, Yao TW, Chowdhury S, Nadvi NA, Osborne B, Church WB, McCaughan GW, Gorrell MD (2010). The dipeptidyl peptidase IV family in cancer and cell biology. FEBS J.

[58] El Khoury F, Corcos L, Durand S, Simon B, Le Jossic-Corcos C (2016). Acquisition of anticancer drug resistance is partially associated with cancer stemness in human colon cancer cells. Int J Oncol.

[59] Cutler MJ, Lowthers EL, Richard CL, Hajducek DM, Spagnuolo PA, Blay J (2015). Chemotherapeutic agents attenuate CXCL12-mediated migration of colon cancer cells by selecting for CXCR4-negative cells and increasing peptidase CD26. BMC Cancer.

[60] Cheung AH, Iyer DN, Lam CS, Ng L, Wong SKM, Lee HS, Wan T, Man J, Chow AKM, Poon RT, Pang R, Law WL (2017). Emergence of CD26+ Cancer Stem Cells with Metastatic Properties in Colorectal Carcinogenesis. Int J Mol Sci.

[61] Liang PI, Yeh BW, Li WM, Chan TC, Chang IW, Huang CN, Li CC, Ke HL, Yeh HC, Wu WJ, Li CF (2017). DPP4/CD26 overexpression in urothelial carcinoma confers an independent prognostic impact and correlates with intrinsic biological aggressiveness. Oncotarget.

[62] Zoller M (2009). Tetraspanins: push and pull in suppressing and promoting metastasis. Nat Rev Cancer.

[63] Greco C, Bralet MP, Ailane N, Dubart-Kupperschmitt A, Rubinstein E, Le Naour F, Boucheix C (2010). E-cadherin/p120-catenin and tetraspanin Co-029 cooperate for cell motility control in human colon carcinoma. Cancer Res.

[64] Heiler S, Wang Z, Zoller M (2016). Pancreatic cancer stem cell markers and exosomes - the incentive push. World J Gastroenterol.

[65] Salvatori L, Caporuscio F, Verdina A, Starace G, Crispi S, Nicotra MR, Russo A, Calogero RA, Morgante E, Natali PG, Russo MA, Petrangeli E (2012). Cell-to-cell signaling influences the fate of prostate cancer stem cells and their potential to generate more aggressive tumors. PLoS One.

[66] He HL, Lee YE, Chen HP, Hsing CH, Chang IW, Shiue YL, Lee SW, Hsu CT, Lin LC, Wu TF, Li CF (2015). Overexpression of DNAJC12 predicts poor response to neoadjuvant concurrent chemoradiotherapy in patients with rectal cancer. Exp Mol Pathol.

[67] De Bessa SA, Salaorni S, Patrao DF, Neto MM, Brentani MM, Nagai MA (2006). JDP1 (DNAJC12/Hsp40) expression in breast cancer and its association with estrogen receptor status. Int J Mol Med.

[68] Sterrenberg JN, Blatch GL, Edkins AL (2011). Human DNAJ in cancer and stem cells. Cancer Lett.

[69] Kok M, Koornstra RH, Margarido TC, Fles R, Armstrong NJ, Linn SC, Van't Veer LJ, Weigelt B (2009). Mammosphere-derived gene set predicts outcome in patients with ER-positive breast cancer. J Pathol.

[70] Kleeberger W, Bova GS, Nielsen ME, Herawi M, Chuang AY, Epstein JI, Berman DM (2007). Roles for the stem cell associated intermediate filament Nestin in prostate cancer migration and metastasis. Cancer Res.

[71] Neradil J, Veselska R (2015). Nestin as a marker of cancer stem cells. Cancer Sci.

[72] Liu T, Xu F, Du X, Lai D, Liu T, Zhao Y, Huang Q, Jiang L, Huang W, Cheng W, Liu Z (2010). Establishment and characterization of multi-drug resistant, prostate carcinoma-initiating stem-like cells from human prostate cancer cell lines 22RV1. Mol Cell Biochem.

[73] Zhang Y, Zeng S, Ma J, Deng G, Qu Y, Guo C, Shen H (2016). Nestin overexpression in hepatocellular carcinoma associates with epithelial-mesenchymal transition and chemoresistance. J Exp Clin Cancer Res.

[74] Kroon J, Kooijman S, Cho NJ, Storm G, van der Pluijm G (2016). Improving Taxane-Based Chemotherapy in Castration-Resistant Prostate Cancer. Trends Pharmacol Sci.

[75] Barallon R, Bauer SR, Butler J, Capes-Davis A, Dirks WG, Elmore E, Furtado M, Kline MC, Kohara A, Los GV, MacLeod RA, Masters JR, Nardone M (2010). Recommendation of short tandem repeat profiling for authenticating human cell lines, stem cells, and tissues. In Vitro Cell Dev Biol Anim.

[76] National Insititues of Health (NIH) Implementing rigor and transparency in NIH & AHRQ Research Grant Applications Notice Number: NOT-OD-16-011.

[77] Yu Y, Fuscoe JC, Zhao C, Guo C, Jia M, Qing T, Bannon DI, Lancashire L, Bao W, Du T, Luo H, Su Z, Jones WD (2014). A rat RNA-Seq transcriptomic BodyMap across 11 organs and 4 developmental stages. Nat Commun.

[78] Team RC (2014). R: A language and environment for statistical computing.

[79] Subramanian A, Tamayo P, Mootha VK, Mukherjee S, Ebert BL, Gillette MA, Paulovich A, Pomeroy SL, Golub TR, Lander ES, Mesirov JP (2005). Gene set enrichment analysis: a knowledge-based approach for interpreting genome-wide expression profiles. Proc Natl Acad Sci U S A.

[80] Mootha VK, Lindgren CM, Eriksson KF, Subramanian A, Sihag S, Lehar J, Puigserver P, Carlsson E, Ridderstrale M, Laurila E, Houstis N, Daly MJ, Patterson N (2003). PGC-1alpha-responsive genes involved in oxidative phosphorylation are coordinately downregulated in human diabetes. Nat Genet.

[81] Ailane N, Greco C, Zhu Y, Sala-Valdes M, Billard M, Casal I, Bawa O, Opolon P, Rubinstein E, Boucheix C (2014). Effect of an anti-human Co-029/tspan8 mouse monoclonal antibody on tumor growth in a nude mouse model. Front Physiol.

