# Supplementary material for: RNA sequencing reveals upregulation of a transcriptomic program associated with stemness in metastatic prostate cancer cells selected for taxane resistance
Source: Oncotarget. 2018 Jul 13;9(54):30363–84. doi: 10.18632/oncotarget.25744 (PMC6084384; doi:10.18632/oncotarget.25744)
Supplement: Supplementary file 1 [file oncotarget-09-30363-s001.pdf]

RNA sequencing reveals upregulation of a transcriptomic program associated with stemness in metastatic prostate cancer cells selected for taxane resistance

SUPPLEMENTARY MATERIALS

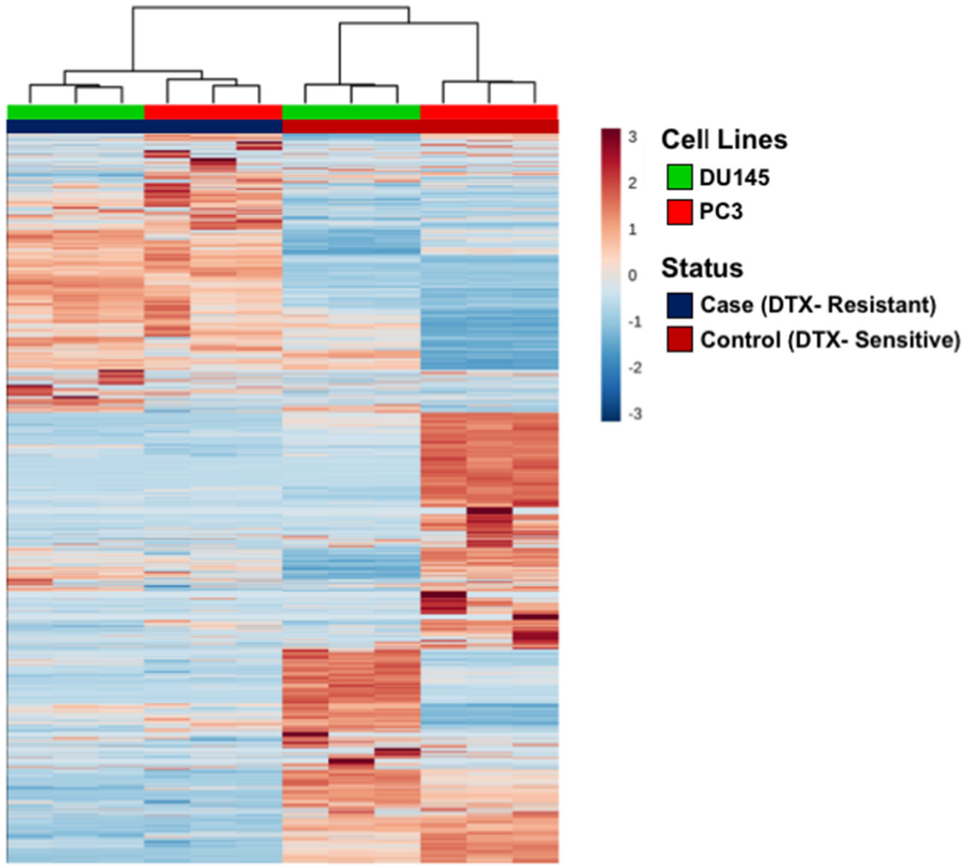

Supplementary Figure 1: Hierarchical clustering heat map of global gene analysis for all cell lines (PC3, DU145, PC3-DR, and DU145-DR).

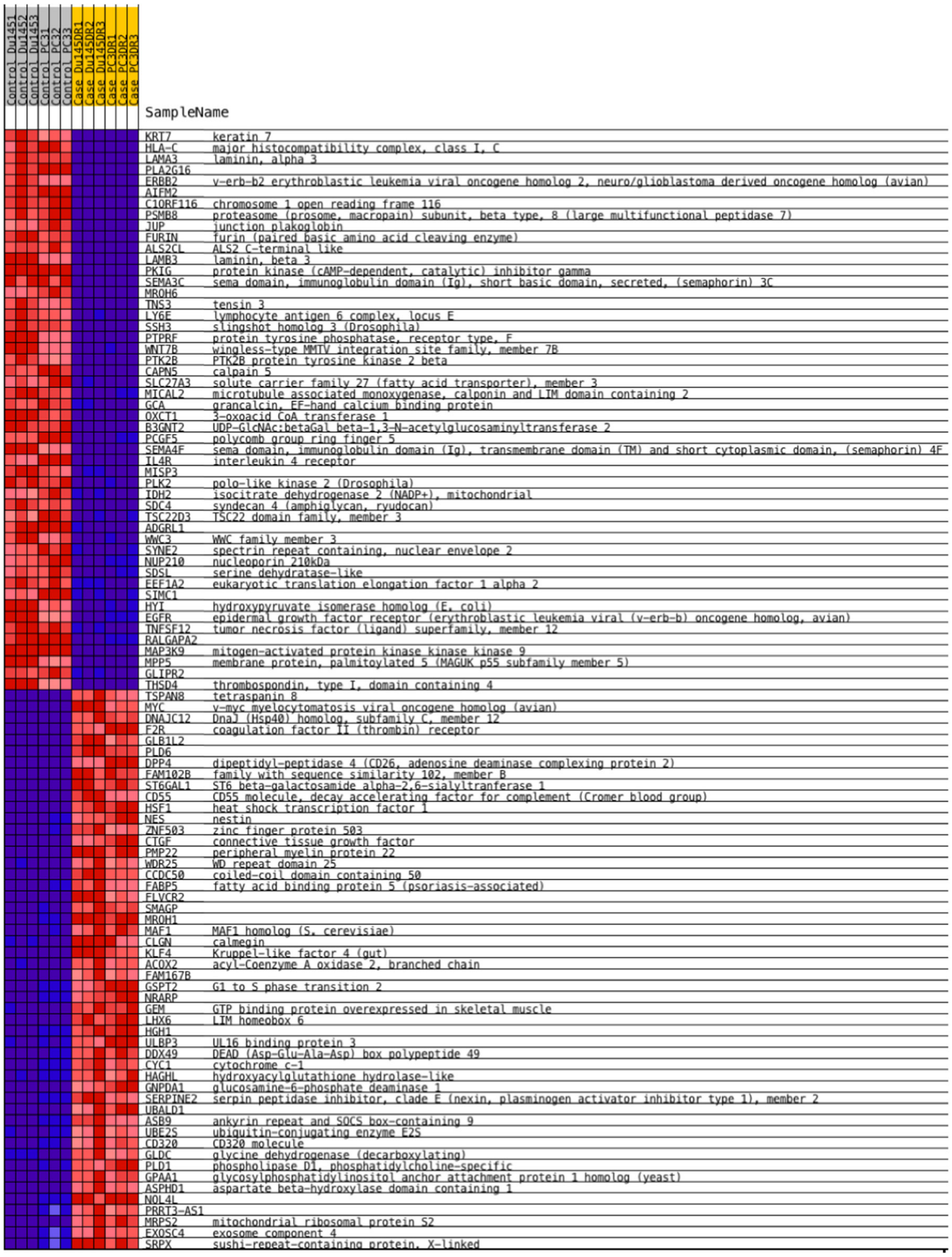

Supplementary Figure 2: GSEA generated heat map of the top ranked 50 overlap genes significantly downregulated (blue) or upregulated (red) between DTX-sensitive PC3 and DU145 compared to DTX-resistant PC3-DR and DU145-DR cells.

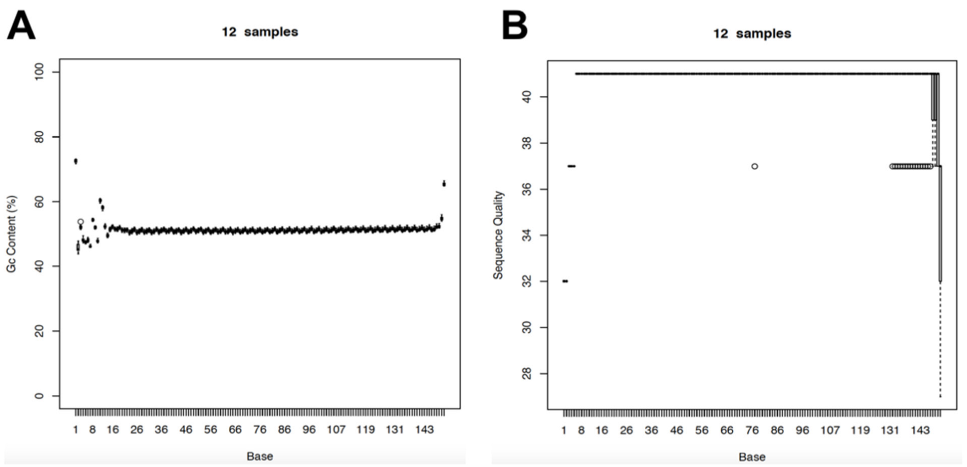

Supplementary Figure 3: Quality assessment metrics for RNA-seq data. Box plots representing interquartile range and median of **(A)** GC content (%) and **(B)** the Phred quality score distribution over all reads across all 12 samples in each base.

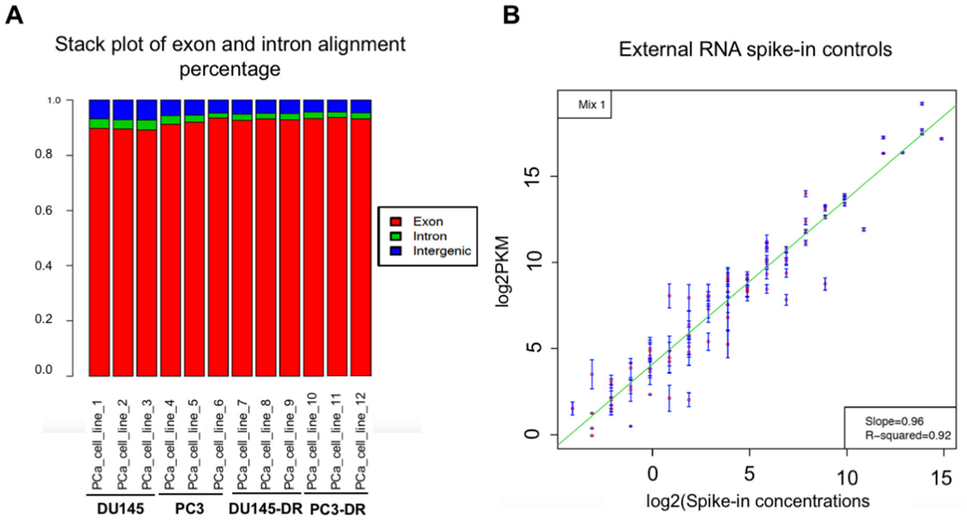

Supplementary Figure 4: Quality assessment on external RNA spike-in controls. (A) Percentage of reads mapped to genomic regions including exon, intron, and intergenic region. **(B)** Plot of log2 (FPKM) of ERCCs detected from samples spiked with ERCC Mix1 vs log^2 (spike-in concentrations).

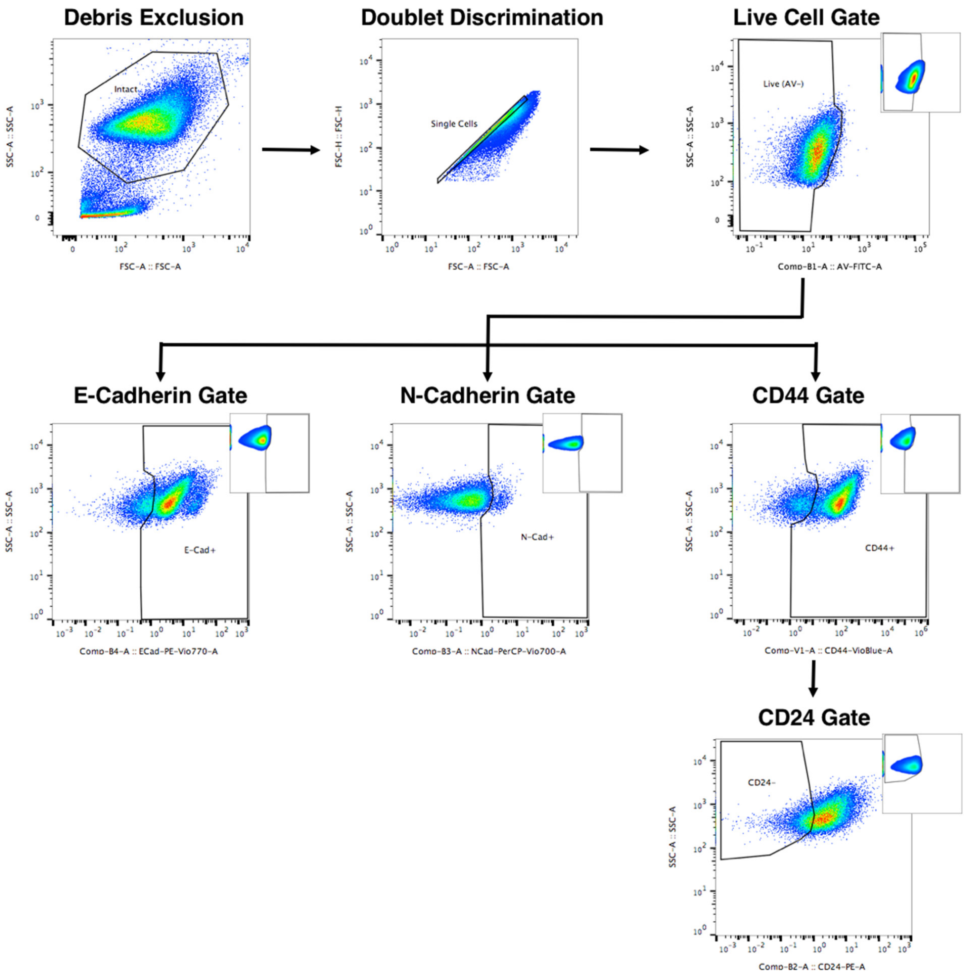

Supplementary Figure 5: Gating strategy for multicolor flow cytometric analysis of CSC markers in cells grown in adherent (2D) or non-adherent (3D) conditions. Following compensation, gates were set for SSC-A versus marker expression based on Fluorescence-Minus-One (FMO) controls (inset).

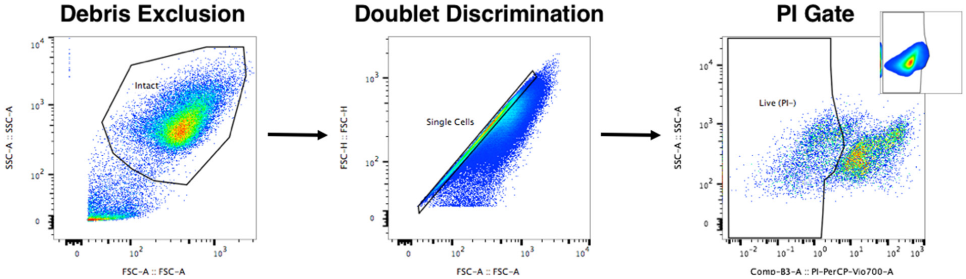

Supplementary Figure 6: Gating strategy for flow cytometric analysis of cell death. Propidium iodide (PI) staining was analyzed in DU145 and DU145-DR cells grown in adherent or non-adherent conditions following 72 hours of exposure to DTX. Gates for PI were set using unstained controls on pooled samples (right inset).

Supplementary Table 1: GSEA top-25 ranked RNA-seq downregulated genes

Gene Name	Gene Title	Rank Score (GSEA)	Log_2 Fold Change PC3 vs. PC3-DR	Log_2 Fold Change DU145 vs. DU145-DR
KRT7	keratin 7	4.702	-7.497	-7.843
HLA-C	major histocompatibility complex, class I, C	4.500	-5.337	-4.225
LAMA3	laminin, alpha 3	4.494	-4.056	-4.376
PLA2G16	phospholipase A2 Group XVI	4.332	-3.905	-4.489
ERBB2	v-erb-b2 erythroblastic leukemia viral oncogene homolog 2, neuro/glioblastoma derived oncogene homolog (avian)	4.267	-3.339	-3.632
AIFM2	apoptosis inducing factor, mitochondria associated 2	4.193	-4.127	-3.373
C1ORF116	chromosome 1 open reading frame 116	4.157	-3.317	-3.529
PSMB8	proteasome subunit beta type, 8 (large multifunctional peptidase 7)	4.133	-6.159	-3.699
JUP	junction plakoglobin	4.100	-4.461	-3.666
FURIN	furin (paired basic amino acid cleaving enzyme)	4.059	-3.102	-3.391
ALS2CL	ALS2 C-terminal like	4.032	-3.975	-3.194
LAM83	laminin, beta 3	3.984	-3.587	-4.206
PKIG	protein kinase (cAMP-dependent, catalytic inhibitor gamma	3.963	-3.214	-2.976
SEMA3C	sema domain, immunoglobin domain (Ig), short basic domain, secreted, (semaphorin) 3C	3.939	-2.957	-3.166
MROH6	maestro heat-like repeat family member 6	3.906	-4.171	-2.881
TNS3	tensin 3	3.858	-2.992	-2.889
LY6E	lymphocyte antigen 6 complex, locus E	3.834	-3.344	-2.945
SSH3	slingshot homolog 3 (Drosophila)	3.801	-2.733	-2.984
PTPRF	protein tyrosine phosphatase, receptor type, F	3.765	-2.463	-3.473
WNT7B	wingless-type MMTV integration site family, member 7B	3.686	-2.694	-4.630
PTK28	PTK2B protein tyrosine kinase 2 beta	3.632	-3.658	-3.552
CAPN5	calpain 5	3.614	-2.852	-2.380
SLC27A3	solute carrier family 27 (fatty acid transporter), member 3	3.592	-3.285	-2.310
MICAL2	microtubule associated monoxygenase, calponin and LIM domain containing 2	3.590	-2.301	-2.910
GCA	grancalcin, EF-hand calcium binding protein	3.575	-3.679	-2.472

Supplementary Table 2: Primer sequences for RNA-seq gene validation using in-house qPCR

Gene	Forward Sequence (5' to 3')	Reverse Sequence (5' to 3')
DPP4	CTCCAGAAGACAACCTTGACCATTACAGAA	TCATCATCATCTTGACAGTGCAGTTTTGAG
TSPAN8	TTGCTTCTGATCCTGCTCCT	TTTTTCACTTTCCCCTGTGG
NES	CTCCAAGAATGGAGGCTGTAGGAA	CCTATGAGATGGAGCAGGCAAGA
DNAJC12	CAGACAAGCATCCTGAAAACCC	TCGCCAGTGGTCATAGCGGGC
FABP5	ACCCTGGGAGAGAAGTTTGAAGA	TGTAAAGTTGCAGACAGTCTGAGTTTT
BOP1	CCATGCCGAGTCTTACAACCCACC	AGCAGCAACACGGCATCATCCATGGC
ABCC3	CTGTGCACACAGAAAACCCG	GGACACCCAGGACCATCTTG
TGM2	TAAGAGATGCTGTGGAGGAG	CGAGCCCTGGTAGATAAA
GAPDH	CGAGATCCCTCCAAAATCAA	TTCACACCCATGACGAACAT

Supplementary Table 3: Antibodies used for detection of EMT and CSC markers by flow cytometry

Antibody	Fluorochrome	Manufacturer Information	Laser
CD44	V450	BD Biosciences<br>Cat# 561292<br>Clone: G44-26	Violet
Annexin-V	FITC	Life Technologies<br>Cat# V13242	
CD24	PE	BD Biosciences<br>Cat# 555428	
N-Cadherin	PE-Vio770	BD Biosciences<br>Cat# 56345<br>Clone: 8C11	Blue
E-Cadherin	PE-Vio770	Miltenyi Biotec<br>Cat# 130-099-142	

Supplementary Table 4: Fluorescence-Minus-One (FMO) staining strategy for detection of CSC and EMT markers by flow cytometry

Antibody	CD44	Annexin-V	CD24	N-Cadherin	E-Cadherin
Unstained Control	-	-	-	-	-
CD44 Only	+	-	-	-	-
Annexin-V Only	-	+	-	-	-
CD24 Only	-	-	+	-	-
N-Cadherin Only	-	-	-	+	-
E-Cadherin Only	-	-	-	-	+
FMO-CD44	-	+	+	+	+
FMO-AV	+	-	+	+	+
FMO-CD24	+	+	-	+	+
FMO-N-Cadherin	+	+	+	-	+
FMO-E-Cadherin	+	+	+	+	-
Full Stain	+	+	+	+	+
